# Blood pressure measurement and adverse pregnancy outcomes: A cohort study testing blood pressure variability and alternatives to 140/90 mmHg

**DOI:** 10.1111/1471-0528.17724

**Published:** 2023-12-06

**Authors:** Milly G. Wilson, Jeffrey N. Bone, Laura J. Slade, Hiten D. Mistry, Joel Singer, Sarah R. Crozier, Keith M. Godfrey, Janis Baird, Peter von Dadelszen, Laura A. Magee

**Affiliations:** ^1^ Department of Women and Children's Health, Faculty of Medicine, School of Life Course and Population Sciences King's College London London UK; ^2^ British Columbia Children's Hospital Research Institute University of British Columbia Vancouver British Columbia Canada; ^3^ Department of Obstetrics and Gynaecology University of British Columbia Vancouver British Columbia Canada; ^4^ Robinson Research Institute The University of Adelaide Adelaide South Australia Australia; ^5^ Department of Obstetrics and Gynaecology Women's and Children's Hospital Adelaide South Australia Australia; ^6^ School of Population and Public Health University of British Columbia Vancouver British Columbia Canada; ^7^ MRC Lifecourse Epidemiology Centre University of Southampton Southampton UK; ^8^ NIHR Applied Research Collaboration Wessex, Southampton Science Park Southampton UK; ^9^ NIHR Southampton Biomedical Research Centre University of Southampton and University Hospital Southampton NHS Foundation Trust Southampton UK

**Keywords:** adverse pregnancy outcomes, American College of Cardiology/American Heart Association guidelines, blood pressure, hypertension, hypertensive disorders of pregnancy, pre‐eclampsia, preterm birth, visit‐to‐visit variability

## Abstract

**Objective:**

To examine the association with adverse pregnancy outcomes of: (1) American College of Cardiology/American Heart Association blood pressure (BP) thresholds, and (2) visit‐to‐visit BP variability (BPV), adjusted for BP level.

**Design:**

An observational study.

**Setting:**

Analysis of data from the population‐based UK Southampton Women's Survey (SWS).

**Population or sample:**

3003 SWS participants.

**Methods:**

Generalised estimating equations were used to estimate crude and adjusted relative risks (RRs) of adverse pregnancy outcomes by BP thresholds, and by BPV (as standard deviation [SD], average real variability [ARV] and variability independent of the mean [VIM]). Likelihood ratios (LRs) were calculated to evaluate diagnostic test properties, for BP at or above a threshold, compared with those below.

**Main outcome measures:**

Gestational hypertension, severe hypertension, pre‐eclampsia, preterm birth (PTB), small‐for‐gestational‐age (SGA) infants, neonatal intensive care unit (NICU) admission.

**Results:**

A median of 11 BP measurements were included per participant. For BP at ≥20 weeks’ gestation, higher BP was associated with more adverse pregnancy outcomes; however, only BP <140/90 mmHg was a good rule‐out test (negative LR <0.20) for pre‐eclampsia and BP ≥140/90 mmHg a good rule‐in test (positive LR >8.00) for the condition. BP ≥160/110 mmHg could rule‐in PTB, SGA infants and NICU admission (positive LR >5.0). Higher BPV (by SD, ARV, or VIM) was associated with gestational hypertension, severe hypertension, pre‐eclampsia, PTB, SGA and NICU admission (adjusted RRs 1.05–1.39).

**Conclusions:**

While our findings do not support lowering the BP threshold for pregnancy hypertension, they suggest BPV could be useful to identify elevated risk of adverse outcomes.

## 1 INTRODUCTION

The hypertensive disorders of pregnancy (HDP) are associated with a substantial global burden of maternal, fetal, and newborn morbidity and mortality. Currently, all international guidelines define hypertension in pregnancy as a systolic blood pressure (sBP) ≥140 mmHg or a diastolic BP (dBP) ≥90 mmHg.^1^

Outside pregnancy, there is a linear relationship between higher BP and heightened cardiovascular risk.^2^ To encourage improvement in clinical outcomes through better BP control, the American College of Cardiology (ACC) and American Heart Association (AHA) revised their definition of hypertension outside pregnancy in 2017. The former threshold of 140/90 mmHg was replaced by a tiered system of: ‘Normal BP’ (sBP <120 mmHg and dBP <80 mmHg); ‘Elevated BP’ (sBP 120–129 mmHg and dBP <80 mmHg); ‘Stage 1 hypertension’ (sBP 130–139 mmHg or dBP 80–89 mmHg); and ‘Stage 2 hypertension’ (sBP ≥140 mmHg or dBP ≥90 mmHg).^3^ The ACC/AHA Task Force on Clinical Practice Guidelines called for investigations into use of these lower BP thresholds in pregnancy. Systematic reviews have disclosed an association between these lower BP thresholds and heightened risk of adverse pregnancy outcomes, for BP values measured either before 20^+0^ weeks’ gestation or at ≥20^+0^ weeks’ gestation.^4^, ^5^ However, none of the BP thresholds <140/90 mmHg demonstrated diagnostic test properties reflective of a useful ‘rule‐out’ or ‘rule‐in’ test for development of adverse pregnancy outcomes.

In addition to higher BP level, higher long‐term visit‐to‐visit BP variability (BPV) is a risk factor for cardiovascular disease outside pregnancy, even when adjusted for BP level.^6^ Six previous studies have explored the relationship between BPV and adverse outcomes in pregnancy; results have been conflicting with regard to a relationship between BPV and adverse maternal and/or perinatal outcomes, and whether higher BPV is predictive of adverse outcomes or represents a manifestation of them.^7^, ^8^, ^9^, ^10^, ^11^, ^12^, ^13^

### 1.1 Aims and objectives

Using data from the population‐based UK Southampton Women's Survey (SWS), we aimed to:

1. Analyse the relationship between ACC/AHA BP thresholds and adverse maternal and perinatal outcomes, as well as the diagnostic test properties of ACC/AHA BP thresholds.
2. Analyse the relationship between visit‐to‐visit BPV and adverse maternal and perinatal outcomes.

## 2 METHODS

### 2.1 Southampton Women's Survey

This is a secondary analysis of data from the SWS, a UK‐based pregnancy cohort for which comprehensive details have been published previously.^14^ In brief, between 1998 and 2002, 12 583 non‐pregnant women living in Southampton were recruited. Of these women, 3158 went on to have singleton pregnancies resulting in live births a median of 1.1 years later.

Women were interviewed preconception (at recruitment) and at 11 and 34 weeks’ gestation. Details were recorded about ethnicity, education, smoking, body mass index (BMI), social deprivation and parity. Pregnancy care and outcomes were abstracted from maternity records by research nurses and included pregnancy hypertension (see below), mode of delivery, postpartum haemorrhage (PPH), fetal sex, gestation at delivery, birthweight and neonatal intensive care unit (NICU) admission.

All clinical antenatal BP measurements were abstracted from maternity records, ordered by time, and checked for accuracy according to protocol.^14^ When multiple readings were recorded at the same visit, the mean was taken as the measurement for that visit. Chronic hypertension was defined as use of antihypertensive medication pre‐pregnancy or at the 11 weeks’ gestation visit, or sBP ≥140 mmHg or dBP ≥90 mmHg on any occasion at <20^+0^ weeks’ gestation. Any diagnoses of gestational hypertension or pre‐eclampsia were accepted, as abstracted from maternity records. Gestational hypertension was defined as sBP ≥140 mmHg or dBP ≥90 mmHg, on any occasion at ≥20^+0^ weeks’ gestation, in a previously normotensive woman. In the UK until 2019, pre‐eclampsia was defined as gestational hypertension with new‐onset proteinuria.^15^ Severe hypertension was derived and classified as sBP ≥160 mmHg or dBP ≥110 mmHg.

All participants provided informed consent and the study was approved by the Southampton and Southwest Hampshire Local Research Ethics Committee (08/H0502/95).

### 2.2 BP measurements

To enable calculation of BPV, we included women with at least three BP measurements in pregnancy.

Each sBP and dBP measurement per visit was categorised according to ACC/AHA criteria, for each of <20^+0^ and ≥20^+0^ weeks’ gestation as: ‘Normal BP’ (sBP <120 mmHg and dBP <80 mmHg), ‘Elevated BP’ (sBP 120–129 mmHg and dBP <80 mmHg), ‘Stage 1 hypertension’ (sBP 130–139 mmHg or dBP 80–89 mmHg) or ‘Stage 2 hypertension’ (sBP ≥140 mmHg or dBP ≥90 mmHg).^16^ ‘Stage 2 hypertension’ was divided into non‐severe ‘Stage 2 hypertension’ (sBP 140–159 mmHg or dBP 90–109 mmHg) and severe ‘Stage 2 hypertension’ (sBP ≥160 mmHg or dBP ≥110 mmHg). The lower category of each pair of consecutive visits was taken as the category for that pair of visits. The category for the gestational period as a whole was taken as the highest overall category. Each participant's mean BP was calculated using all BP values available, to be used for adjustment of BPV, as higher BP levels are associated with more BPV and adverse pregnancy outcomes.^17^

BPV was defined as visit‐to‐visit, using three traditional measures of variability: (i) within‐participant standard deviation (SD), to reflect dispersion of BP measurements around mean BP; (ii) average real variability (ARV), as the average of absolute successive differences between BPs, reflecting changes over short periods of time; and (iii) variability independent of the mean (VIM), derived from non‐linear regression analysis and able to differentiate from effects of mean BP. Formulas used for each measure are available in Table S1.

### 2.3 Outcomes

Key outcomes were the HDP, preterm birth (PTB, at <37^+0^ weeks’ gestation), small‐for‐gestational‐age infants (SGA, as birthweight <10th centile for gestational age and sex, by Intergrowth‐21st standards)^18^ and NICU admission. HDP were accepted as abstracted by the SWS team, even if the supporting BP values were not also abstracted.

Core maternal outcomes in pregnancy hypertension that were not available from maternity records included: maternal mortality, eclampsia, stroke, blindness, retinal detachment, pulmonary edema, kidney injury, liver capsule haematoma/rupture, placental abruption, raised liver enzymes, intensive care unit (ICU) admission, intubation and mechanical ventilation.^19^ Offspring outcomes not analysed because of low prevalence in SWS included stillbirth, neonatal mortality, neonatal seizures and neonatal respiratory support.^19^

### 2.4 Statistical analysis

Descriptive analyses were undertaken for baseline maternal characteristics, BP thresholds, BPV and maternal and perinatal outcomes.

To assess the relationship between BP thresholds and adverse outcomes, we used Poisson models with robust variance to estimate the crude and adjusted risk ratios (aRRs) between ‘Normal BP’ and each ACC/AHA BP threshold and each outcome. To assess the diagnostic test properties of these cut‐points we calculated sensitivity, specificity, positive likelihood ratios (+LR, as sensitivity/[1 – specificity]) and negative LR (−LR, as [1 – sensitivity]/specificity), using the lower limit of each category as a cut‐off for abnormal BP; this is the current clinical methodology for comparing women with BP ≥140/90 mmHg (versus BP below this threshold). Based on point estimates, +LR ≥5.0 and −LR ≤0.2 were interpreted as ‘good’.^20^ To assess the relationship between BPV and adverse outcomes, we again used Poisson models with robust variance to estimate crude and aRRs for each measure of BPV, for sBP and dBP separately. All Poisson models included fixed effects for mean BP, maternal age, BMI, parity and smoking status, which were chosen a priori as potential confounders based on previous literature.

For BPV, Spearman correlation (*r*) was explored between the number of BP measurements and each measure of BPV.

In sensitivity analyses, we first explored potential reverse causality (by which BPV may be an artefact of the adverse outcomes themselves), calculating BPV by removing BP values that were within 1, 2, 4 or 6 weeks before birth. Secondly, we omitted participants with chronic hypertension, to examine the impact of chronic hypertension on the association between BPV and outcomes. Thirdly, we restricted analyses to participants with chronic hypertension, for direct comparison with prior work.^9^

Multiple imputation (generating 50 imputed datasets) was used to address missing data, using the Multivariate Imputation by Chained Equations (MICE) package in R statistical software,^21^ which was used for all data analyses. Imputation models included all prognostic variables and outcomes and results were pooled using Rubin's rules.^22^

For all analyses, results are presented as effect estimates and corresponding 95% confidence intervals.

## 3 RESULTS

### 3.1 Participants

Of the 3158 women in the SWS who delivered a live, singleton baby between 1998 and 2007, 3003 (95.2%) women had at least three BP measurements during pregnancy and were included in this analysis.

Table 1 presents participants’ baseline characteristics and pregnancy outcomes, stratified by ACC/AHA BP category; 38.3% had ‘Normal BP’, 27.1% had ‘Elevated BP’, 25.6% had ‘Stage 1 hypertension’ and 9% had ‘Stage 2 hypertension’ (8.4% non‐severe, 0.6% severe).

**TABLE 1:** Participant characteristics, stratified by ACC/AHA BP criteria: *n* (%)/median [IQR]/mean (SD).^a^

Levels	Total	Normal	Elevated	Stage 1	Stage 2 (non‐severe)	Stage 2 (severe)	Missing *n* (%)
Total, *n* (%)	3003	1150 (38.3)	813 (27.1)	770 (25.6)	252 (8.4)	18 (0.6)	0 (0)
Maternal baseline characteristics							
Maternal age	30.6 (3.8)	30.6 (3.8)	30.4 (3.8)	30.8 (3.9)	30.9 (3.7)	30.0 (4.3)	0 (0)
Ethnicity							
White	2866 (95.4)	1073 (93.3)	774 (95.2)	753 (97.8)	248 (98.4)	18 (100.0)	0 (0)
Black	23 (0.8)	13 (1.1)	8 (1.0)	0 (0.0)	2 (0.8)	0 (0.0)	
Asian	97 (3.2)	56 (4.9)	26 (3.2)	14 (1.8)	1 (0.4)	0 (0.0)	
Other	17 (0.6)	8 (0.7)	5 (0.6)	3 (0.4)	1 (0.4)	0 (0.0)	
Early pregnancy BMI (kg/m^2^)	24.9 [22.6–28.5]	23.5 [21.5–26.0]	25.0 [22.8–27.6]	27.1 [24.0–31.0]	27.4 [24.3–32.1]	29.5 [25.5–35.8]	932 (31.0)
Pregnancy weight gain (kg)	12.2 (6.2)	11.1 (5.2)	12.1 (5.9)	12.7 (6.9)	14.8 (7.8)	20.1 (10.4)	925 (30.8)
Nulliparous	1522 (50.7)	524 (45.6)	401 (49.3)	422 (54.8)	162 (64.3)	13 (72.2)	0 (0)
Smoking	471 (15.7)	188 (16.3)	130 (16.0)	118 (15.3)	34 (13.5)	1 (5.6)	140 (4.7)
Pregestational diabetes	12 (0.4)	4 (0.3)	0 (0.0)	5 (0.6)	2 (0.8)	1 (5.6)	294 (9.8)
Pre‐existing hypertension	68 (2.3)	8 (0.7)	10 (1.2)	26 (3.4)	22 (8.7)	2 (11.1)	0 (0)
Antihypertensive treatment at 11 weeks’ gestation	12 (0.4)	1 (0.1)	0 (0.0)	3 (0.4)	6 (2.4)	2 (11.1)	0 (0)
Maternal pregnancy outcomes							
Chronic hypertension	213 (7.1)	19 (1.7)^b^	21 (2.6)	95 (12.3)	70 (27.8)	8 (44.4)	52 (1.7)
Gestational hypertension	144 (4.8)	6 (0.5)^c^	9 (1.1)^c^	46 (6.0)^c^	75 (29.8)	8 (44.4)	0 (0)
Preeclampsia	88 (2.9)	4 (0.3)^c^	5 (0.6)^c^	22 (2.9)^c^	46 (18.3)	11 (61.1)	0 (0)
Gestational diabetes mellitus	37 (1.2)	9 (0.8)	8 (1.0)	15 (1.9)	4 (1.6)	1 (5.6)	0 (0)
Severe hypertension	86 (2.9)	2 (0.2)	5 (0.6)	10 (1.3)	51 (20.2)	18 (100.0)	0 (0)
Labour induction	861 (28.7)	295 (25.7)	198 (24.4)	231 (30.0)	119 (47.2)	18 (100.0)	0 (0)
Caesarean	717 (23.9)	228 (19.8)	197 (24.2)	196 (25.5)	84 (33.3)	12 (66.7)	0 (0)
GA at delivery, weeks	40.0 [39.1–41.0]	39.9 [38.9–40.9]	40.1 [39.2–41.0]	40.1 [39.1–41.1]	40.3 [39.1–41.1]	38.5 [36.8–40.1]	0 (0)
Neonatal characteristics							
Stillbirth	0 (0)	0 (0)	0 (0)	0 (0)	0 (0)	0 (0)	0 (0)
Neonatal death	0 (0)	0 (0)	0 (0)	0 (0)	0 (0)	0 (0)	0 (0)
PTB	167 (5.6)	78 (6.8)	25 (3.1)	39 (5.1)	20 (7.9)	5 (27.8)	0 (0)
SGA	169 (5.6)	67 (5.8)	37 (4.6)	34 (4.4)	26 (10.3)	5 (27.8)	0 (0)
NICU admission	183 (6.1)	70 (6.1)	32 (3.9)	53 (6.9)	23 (9.1)	5 (27.8)	7 (0.2)

Abbreviations: ACC, American College of Cardiology; AHA, American Heart Association; BMI, body mass index; BP, blood pressure; GA, gestational age; NICU, neonatal intensive care unit; PTB, preterm birth; SGA, small for gestational age.

^a^ *χ* ^2^ test was used for categorical variables, and the Kruskal–Wallis test/ANOVA for continuous ones.

^b^ We included women using antihypertensive therapy at 11 weeks' and women with a pre‐pregnancy diagnosis of hypertension to define chronic hypertension – 19 of these participants had normal BP.

^c^ Gestational hypertension and preeclampsia were not derived using ACC/AHA criteria but taken from the women's obstetric records. Gestational hypertension was modified according to chronic hypertension classification.

Most women were around 30 years old, of white ethnicity, nulliparous, and non‐smokers (Table 1). Most baseline characteristics varied by BP category; higher BP level in pregnancy was associated with white ethnicity, higher early pregnancy BMI, higher pregnancy weight gain, nulliparity, chronic hypertension and early pregnancy antihypertensive therapy specifically.

Birth occurred at about 40 weeks’ gestation in each BP category (Table 1). Just over one‐quarter of women were induced and just under one‐quarter were delivered by caesarean section. Almost 8% of women developed either gestational hypertension or pre‐eclampsia. There were 61 women with gestational hypertension and 31 with pre‐eclampsia whose maximal BP in pregnancy was <140/90 mmHg but whose diagnoses were abstracted by the SWS from maternity records and not derived using ACC/AHA criteria. The incidence of pregnancy complications generally increased with higher BP category.

### 3.2 BP characteristics

Participants had a median of 11.0 BP measurements during pregnancy (Table S2), most at ≥20 weeks’ gestation. Median BP level during pregnancy was 112.0/68.5 mmHg. Median sBP/dBP variability was 8.2/6.6 mmHg by SD, 8.2/6.6 by VIM, and 7.5/5.8 mmHg by ARV. Both BP level and BPV appeared lower at <20 than ≥20 weeks’ gestation.

For maximum BP at <20 weeks’ gestation, half of women with ‘Normal BP’ (1090 [51.4%] of 2122 women) or ‘Elevated BP’ (261 [50.3%] of 519 women) had higher BP in the second half of pregnancy (Table S3). In contrast, most women with ‘Stage 1 hypertension’ (185 [70.3%] of 263 women) or ‘Non‐severe Stage 2 hypertension’ (40 [97.6%] of 41 women) had BP that did not rise further in the second half of pregnancy.

There was a greater percentage change in BP from booking in the first 20 weeks in women who developed adverse outcomes, compared with those who did not (Table S4).

There were minimal relationships between the number of BP measurements and BPV, measured by SD, ARV or VIM (correlation coefficients ≤0.29; Table S5).

### 3.3 BP level and pregnancy outcomes

Compared with ‘Normal BP’, all higher BP categories were associated with pre‐eclampsia for maximum BP either before or after 20 weeks’ gestation (Table 2). Otherwise at <20 weeks’ gestation, there was a dose–response relationship between higher risk of PTB, SGA and NICU admission; although estimates for severe ‘Stage 2 hypertension’ could not be computed. At ≥20 weeks’ gestation, there was again a dose–response relationship between increasing BP group and outcomes, with RRs generally higher than their counterparts at <20 weeks’. Infants born to women with severe ‘Stage 2 hypertension’ had particularly higher risk of PTB, SGA and NICU admission (all aRRs >3.9).

**TABLE 2:** Relationship between ACC/AHA BP thresholds and adverse pregnancy outcomes.^a^

		<20^+0^ weeks	≥20^+0^ weeks
Adjusted RRs for ACC/AHA BP thresholds and adverse pregnancy outcomes			
Pre‐eclampsia			
Normal BP		Ref	Ref
Elevated BP	Adjusted	1.64 (1.07–2.53)	10.26 (3.77–27.91)
	Crude	1.97 (1.30–2.98)	13.03 (4.79–35.45)
Stage 1 hypertension	Adjusted	2.72 (1.65–4.50)	13.58 (6.77–27.28)
	Crude	3.24 (2.06–5.11)	16.57 (8.35–32.89)
Non‐severe Stage 2 hypertension	Adjusted	2.61 (1.11–6.10)	14.77 (9.51–22.95)
	Crude	4.17 (1.78–9.77)	18.61 (12.24–28.31)
Severe Stage 2 hypertension	Adjusted	0 (0–0)	13.71 (8.46–22.24)
	Crude	0 (0–0)	23.69 (15.42–36.4)
PTB			
Normal BP		Ref	Ref
Elevated BP	Adjusted	1.10 (0.78–1.54)	0.70 (0.52–0.96)
	Crude	1.07 (0.77–1.48)	0.71 (0.53–0.95)
Stage 1 hypertension	Adjusted	1.94 (1.29–2.91)	1.20 (0.88–1.65)
	Crude	1.79 (1.21–2.64)	1.17 (0.87–1.59)
Non‐severe Stage 2 hypertension	Adjusted	2.21 (0.96–5.08)	1.83 (1.20–2.80)
	Crude	2.22 (0.96–5.15)	1.78 (1.19–2.68)
Severe Stage 2 hypertension	Adjusted	0 (0–0)	5.43 (2.44–12.07)
	Crude	0 (0–0)	5.12 (2.39–10.95)
SGA			
Normal BP		Ref	Ref
Elevated BP	Adjusted	1.30 (0.92–1.83)	1.02 (0.74–1.40)
	Crude	1.16 (0.84–1.59)	0.94 (0.70–1.27)
Stage 1 hypertension	Adjusted	2.10 (1.39–3.17)	1.28 (0.93–1.75)
	Crude	1.78 (1.20–2.62)	1.18 (0.87–1.59)
Non‐severe Stage 2 hypertension	Adjusted	1.42 (0.47–4.29)	2.44 (1.67–3.57)
	Crude	1.25 (0.42–3.76)	2.27 (1.57–3.29)
Severe Stage 2 hypertension	Adjusted	0 (0–0)	6.38 (2.72–14.95)
	Crude	0 (0–0)	5.06 (2.36–10.81)
NICU admission			
Normal BP		Ref	Ref
Elevated BP	Adjusted	1.21 (0.89–1.64)	0.91 (0.67–1.24)
	Crude	1.28 (0.95–1.72)	1.00 (0.75–1.33)
Stage 1 hypertension	Adjusted	1.49 (1.00–2.22)	1.39 (1.03–1.88)
	Crude	1.59 (1.08–2.33)	1.50 (1.13–1.98)
Non‐severe Stage 2 hypertension	Adjusted	1.65 (0.73–3.76)	1.65 (1.11–2.47)
	Crude	1.94 (0.84–4.48)	1.83 (1.25–2.68)
Severe Stage 2 hypertension	Adjusted	0 (0–0)	3.97 (1.71–9.21)
	Crude	0 (0–0)	4.65 (2.18–9.94)

Abbreviations: BP, blood pressure; dBP, diastolic blood pressure; NICU, neonatal intensive care unit; PTB, preterm birth; RRs, relative risks; sBP, systolic blood pressure; SGA, small for gestational age.

^a^ BP is categorised as: ‘Normal BP’ (sBP <120 mmHg and dBP <80 mmHg), ‘Elevated BP’ (sBP 120–129 mmHg and dBP <80 mmHg), ‘Stage 1 hypertension’ (sBP 130–139 mmHg or dBP 80–89 mmHg, or both) and ‘Stage 2 hypertension’ (sBP ≥140 mmHg or dBP ≥90 mmHg, or both), including non‐severe ‘Stage 2 hypertension’ (sBP 140–159 mmHg or dBP 90–109 mmHg, or both) and severe ‘Stage 2 hypertension’ (sBP ≥160 mmHg or dBP ≥110 mmHg, or both). All analyses were adjusted for maternal age, body mass index, parity and smoking status. Data are RR (95% CI).

At <20 weeks’ gestation, for the diagnostic test properties of BP, no threshold was useful as a rule‐in (+LRs <5.0) or rule‐out (−LRs >0.20) test for any outcome examined (Table 3). At ≥20 weeks’ gestation, BP consistently <130/80 mmHg was reassuring (a good rule‐out test) for development of pre‐eclampsia, BP ≥140/90 mmHg was a good rule‐in test for development of pre‐eclampsia, and BP ≥160/110 mmHg was a good rule‐in test for PTB, SGA and NICU admission (Table 3, with corresponding sensitivities and specificities in Table S6).

**TABLE 3:** Positive and negative likelihood ratios for ACC/AHA BP categories and pregnancy outcomes (<20/≥20 weeks’ gestation).^a^

	Events, *n* (%)^b^	Positive LR (95% CI)	Negative LR (95% CI)
<20 weeks’ gestation			
Pre‐eclampsia			
Normal BP	50 (2.35)	Ref	Ref
Elevated BP	14 (2.69)	1.57 (1.23, 2.01)	0.78 (0.65, 0.94)
Stage 1 hypertension	19 (7.22)	2.78 (1.94, 3.98)	0.81 (0.71, 0.92)
Non‐severe Stage 2 hypertension	5 (12.2)	4.40 (1.77, 10.92)	0.96 (0.91, 1.01)
Severe Stage 2 hypertension	0 (0)	0.00 (0.00, NaN)	1.00 (1.00, 1.00)
PTB <37 weeks			
Normal BP	117 (5.50)	Ref	Ref
Elevated BP	20 (3.85)	1.04 (0.82, 1.33)	0.98 (0.89, 1.09)
Stage 1 hypertension	23 (8.75)	1.71 (1.20, 2.44)	0.92 (0.86, 0.99)
Non‐severe Stage 2 hypertension	5 (12.2)	2.28 (0.91, 5.72)	0.98 (0.96, 1.01)
Severe Stage 2 hypertension	0 (0)	0.00 (0.00, NaN)	1.00 (1.00, 1.00)
SGA			
Normal BP	116 (5.46)	Ref	Ref
Elevated BP	23 (4.42)	1.10 (0.87, 1.39)	0.96 (0.87, 1.07)
Stage 1 hypertension	25 (9.51)	1.68 (1.18, 2.40)	0.92 (0.86, 0.99)
Non‐severe Stage 2 hypertension	3 (7.32)	1.28 (0.40, 4.10)	1.00 (0.98, 1.02)
Severe Stage 2 hypertension	0 (0)	0.00 (0.00, NaN)	1.00 (1.00, 1.00)
NICU admission			
Normal BP	121 (5.69)	Ref	Ref
Elevated BP	32 (6.15)	1.20 (0.97, 1.49)	0.92 (0.83, 1.03)
Stage 1 hypertension	23 (8.75)	1.55 (1.08, 2.21)	0.94 (0.88, 1.00)
Non‐severe Stage 2 hypertension	5 (12.2)	2.06 (0.82, 5.18)	0.99 (0.96, 1.01)
Severe Stage 2 hypertension	0 (0)	0.00 (0.00, NaN)	1.00 (1.00, 1.00)
≥20 weeks’ gestation			
Pre‐eclampsia			
Normal BP	4 (0.35)	Ref	Ref
Elevated BP	5 (0.62)	1.57 (1.49–1.66)	0.12 (0.04–0.30)
Stage 1 hypertension	22 (2.86)	2.72 (2.49–2.97)	0.15 (0.08–0.28)
Non‐severe Stage 2 hypertension	46 (18.25)	8.86 (7.25–10.84)	0.38 (0.29–0.50)
Severe Stage 2 hypertension	11 (61.1)	52.05 (20.67–131.09)	0.88 (0.81–0.95)
PTB <37 weeks			
Normal BP	78 (6.78)	Ref	Ref
Elevated BP	25 (3.08)	0.86 (0.74–0.99)	1.24 (1.04–1.46)
Stage 1 hypertension	39 (5.06)	1.11 (0.91–1.36)	0.94 (0.83–1.06)
Non‐severe Stage 2 hypertension	20 (7.94)	1.73 (1.18–2.54)	0.93 (0.87–0.99)
Severe Stage 2 hypertension	5 (27.7)	6.53 (2.36–18.10)	0.97 (0.95–1.00)
SGA			
Normal BP	67 (5.83)	Ref	Ref
Elevated BP	37 (4.55)	0.98 (0.86–1.11)	1.04 (0.86–1.26)
Stage 1 hypertension	34 (4.42)	1.12 (0.92–1.36)	0.94 (0.83–1.06)
Non‐severe Stage 2 hypertension	26 (10.32)	2.18 (1.55–3.06)	0.89 (0.83–0.96)
Severe Stage 2 hypertension	5 (27.7)	6.45 (2.33–17.88)	0.97 (0.95–1.00)
NICU admission			
Normal BP	70 (6.09)	Ref	Ref
Elevated BP	32 (3.94)	1.00 (0.89–1.12)	1.00 (0.83–1.21)
Stage 1 hypertension	53 (6.88)	1.30 (1.10–1.54)	0.84 (0.74–0.96)
Non‐severe Stage 2 hypertension	23 (9.13)	1.78 (1.24–2.55)	0.93 (0.87–0.99)
Severe Stage 2 hypertension	5 (27.7)	5.91 (2.13–16.40)	0.98 (0.95–1.00)

Abbreviations: ACC, American College of Cardiology; AHA, American Heart Association; BP, blood pressure; CI, confidence interval; LR, likelihood ratio; NaN, not a number; NICU, neonatal intensive care unit; PTB, preterm birth; SGA, small for gestational age.

^a^ BP is categorised as: ‘Normal BP’ (sBP <120 mm Hg and dBP <80 mm Hg), ‘Elevated BP’ (sBP 120–129 mm Hg and dBP <80 mm Hg), ‘Stage 1 hypertension’ (sBP 130–139 mm Hg or dBP 80–89 mm Hg, or both) and ‘Stage 2 hypertension’ (sBP ≥140 mm Hg or dBP ≥90 mm Hg, or both), including non‐severe ‘Stage 2 hypertension’ (sBP 140–159 mm Hg or dBP 90–109 mm Hg, or both) and severe ‘Stage 2 hypertension’ (sBP ≥160 mm Hg or dBP ≥110 mm Hg, or both). All analyses were adjusted for maternal age, body mass index, parity and smoking status. A positive LR ≥5.00 or a negative LR <0.20 was considered good.

^b^ Events only include women in the category specified; the denominator is women with complete outcome data.

### 3.4 BPV and pregnancy outcomes

**FIGURE 1:**
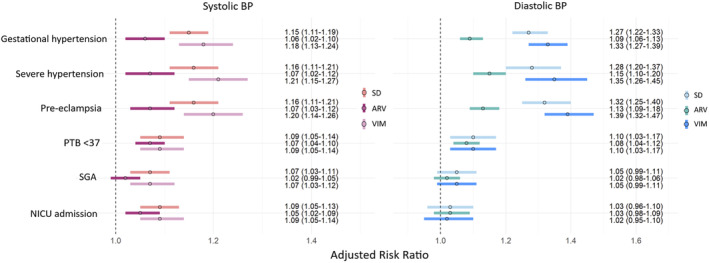
Association between visit‐to‐visit systolic and diastolic BP variability and pregnancy outcomes. ARV, average real variability; BMI, body mass index; BP, blood pressure; dBP, diastolic blood pressure; NICU, neonatal intensive care unit; PTB, preterm birth; sBP, systolic blood pressure; SD, standard deviation; SGA, small for gestational age; VIM, variability independent of the mean. Models are adjusted for maternal age, maternal BMI, parity, sBP or dBP level and smoking status. Numbers represent adjusted relative risks and 95% confidence intervals.

Higher BPV was associated with increased risk of gestational hypertension, severe hypertension, pre‐eclampsia and PTB (see Figure 1 and Table S7 for numeric presentation). This was particularly true for BPV defined by SD and VIM, more than for ARV. Associations were stronger for maternal than perinatal outcomes, but SD and VIM measures of systolic BP variability were still consistent with modest increases in risk of SGA and NICU admission.

The findings for BPV were similar in sensitivity analyses. Progressive removal of BP values from 1 to 6 weeks before birth attenuated the relationships between higher BPV and more PTB, SGA, and NICU admissions; however, the relationships between diastolic BPV, assessed by SD, ARV or VIM, and more hypertension and pre‐eclampsia remained (Table S8). Following exclusion of the 213 women with chronic hypertension, the relationship between higher BPV and more adverse pregnancy outcomes was similar for all outcomes (Table S9). In restricting the analysis to the 213 women with chronic hypertension, higher BPV remained strongly associated with more severe hypertension and pre‐eclampsia (Table S9).

## 4 DISCUSSION

### 4.1 Summary of findings

In the SWS, just over 60% of women had an abnormal BP in pregnancy by ACC/AHA criteria. Higher ACC/AHA BP category and higher BPV were both associated with an increased risk of adverse pregnancy outcomes, following adjustment for prognostic factors.

In general, higher BP (versus ‘Normal BP’) was more strongly associated with adverse pregnancy outcomes. Despite these associations, there was no BP threshold at <20 weeks’ gestation that could usefully reassure or raise the level of concern about pre‐eclampsia or other adverse outcomes. At ≥20 weeks’ gestation, BP <130/80 mmHg could rule‐out development of pre‐eclampsia, BP ≥140/90 mmHg was a good rule‐in test for development of pre‐eclampsia, and BP ≥160/110 mmHg was a good rule‐in test for PTB, SGA and NICU admission.

In addition, higher BPV (adjusted for mean BP and adverse prognostic factors) was associated with more adverse pregnancy outcomes, particularly for BPV defined by SD or VIM and for maternal outcomes (severe hypertension and pre‐eclampsia). Removal of BP values up to 6 weeks before delivery did not attenuate the association between BPV and either severe hypertension or pre‐eclampsia.

### 4.2 Interpretation and comparison with literature

In our meta‐analysis (23 studies, 734 377 women), a BP threshold ≥140/90 mmHg was useful to rule‐in development of pre‐eclampsia (positive LRs ≥5.0),^4^ eclampsia, stroke or maternal ICU admission, consistent with an increased risk of adverse pregnancy outcomes associated with chronic hypertension.^1^ In the present study, at <20 weeks’ gestation, a BP ≥140/90 was just below the threshold for being useful as a diagnostic test for pre‐eclampsia (+LR = 4.40). Our method of defining hypertension using the ACC/AHA criteria was based on consecutive outpatient visits, consistent with clinical care recommendations; in contrast, most studies in the systematic review relied on the single highest BP reading, potentially overestimating the performance of BP ≥140/90 mmHg.

Our finding that at ≥20 weeks’ gestation, the 130/80 mmHg threshold meaningfully reduced the risk of pre‐eclampsia is more reassuring than reported in our systematic review of BP thresholds at ≥20 weeks’ gestation (12 studies, 251 172 women), in which we found a BP ≥140/90 mmHg could meaningfully increase the risk of pre‐eclampsia.^5^ Again, this is likely due to our use of the consecutive BP categorisation method.

As such, based on the diagnostic test properties of BP in pregnancy, we do not recommend lowering the BP threshold for diagnosis of either chronic hypertension in the first half of pregnancy, or gestational hypertension in the second. A BP of ≥140/90 mmHg is useful in identifying pregnancies at increased risk and there is now high‐quality trial evidence that controlling that BP with antihypertensive therapy is beneficial, without increasing risk to the baby.^23^, ^24^

Our finding that higher BPV is associated with more adverse pregnancy outcomes is consistent with some of the prior, limited literature. The International Control of Hypertension In Pregnancy Study (CHIPS) trial (913 pregnancies) of women with chronic or gestational hypertension, showed that higher BPV was associated with more pre‐eclampsia and severe hypertension; however, the associations were likely attributable to BPV manifesting as an artefact of the outcomes themselves. Also, dBP variability may have been associated with fewer adverse perinatal outcomes.^9^ Among 17 770 pregnancies in the Community‐Level Interventions in Pre‐eclampsia (CLIP) trial in Asia and Africa, higher BPV was associated with increased odds of developing hypertension and composite maternal and perinatal death and morbidity. While there was some evidence of reverse causality for maternal outcomes, associations remained between higher BPV and adverse outcomes, and the direction of effect was the same for maternal and perinatal outcomes.^12^ Analyses within the hypertensive subpopulation (as in CHIPS) confirmed an association between higher BPV and more adverse maternal and perinatal outcomes. Furthermore, two large publications (101 100 total participants) have found an association between higher BPV and more SGA infants, with mixed results for other perinatal outcomes.^10^, ^11^ In another publication that included 14 702 women in South Korea, BPV (by SD) was strongly associated with the development of both gestational hypertension and pre‐eclampsia.^8^

While our finding of a stronger association of BPV with maternal (versus perinatal) outcomes is consistent with prior literature, it is possible that the potentially protective effect on perinatal outcomes in CHIPS may have been related to BP control; in the CHIPS trial, women were randomised to ‘tight’ versus ‘less tight’ BP control, whereas contemporaneous BP control in the SWS (1998–2002) and the CLIP trials (by WHO guidance) favoured ‘less tight’ BP control.^25^, ^26^

### 4.3 Strengths and limitations

Strengths of our study include the evaluation of diagnostic test properties of BP level, to provide direct information about the clinical utility of BP thresholds. We adopted commonly used metrics of BPV (SD, ARV and VIM) as in prior publications and adjusted for prognostic factors and mean BP.^6^

Limitations of our study include the modest sample size. Women in SWS were primarily white, limiting the generalisability of our findings to ethnically diverse populations. SWS data are from 1998–2002, with possible differences from contemporary populations in lifestyle factors and prenatal care. Data were restricted to women with singleton pregnancies and live births, and the sample size precluded assessment of the impact of BP level or BPV on perinatal mortality or in multiple pregnancies. BP measurement in the SWS was not standardised, as values were recorded as part of routine antenatal care; while we acknowledge the potential for less measurement precision, an association was still observed between BPV and adverse outcomes, and the BP values included in the analysis reflect real‐world clinical practice. Not all BP measurements for the diagnosis of pregnancy hypertension had been abstracted from maternity records. Similarly, there were no universal measurements of proteinuria, and the definition of pre‐eclampsia at data collection was traditional, based on gestational hypertension and proteinuria. We did not have the date of diagnosis for pregnancy outcomes, and so our sensitivity analyses of BPV‐outcome relationships were based on time of birth;^27^ while the findings of gestational hypertension and severe hypertension are most vulnerable to the limitation of using birthdate for reverse causality assessment, findings were similar to those for pre‐eclampsia. We were not able to adjust for the effect of duration, type, or dose of antihypertensive medication.

## 5 CONCLUSION

Adverse pregnancy outcomes are related to higher BP level and BPV. Our findings support ongoing use of BP ≥140/90 mmHg to define hypertension in maternity care, but also suggest that BPV could serve as a further practical tool for accurate risk stratification. Future work could assess the merits of utilising BP prospectively, calculating BPV at each antenatal care contact, and whether it could function as an additional variable in multivariable prediction models that use combinations of maternal history, biomarkers and ultrasonography to predict the occurrence of placental diseases of pregnancy.^28^ This approach may further progress towards optimising clinical use of BP measurement, to better identify women and babies at risk.

## AUTHOR CONTRIBUTIONS

JNB, LS, HDM, JS, PvD and LAM designed the study. SRC, JNB and KMG curated the data. MGW was responsible for data analysis with support from JNB. All authors approved the final paper for submission and contributed to preparation and editing.

## FUNDING INFORMATION

M. G. Wilson was funded by the KCL Centre for Doctoral Training in Data‐Driven Health (ST12512). The PRECISE Network is funded by the UK Research and Innovation Grand Challenges Research Fund GROW Award scheme (MR/P027938/1). KMG is supported by the UK Medical Research Council (MC_UU_12011/4), the National Institute for Health Research (NIHR Senior Investigator [NF‐SI‐0515‐10042] and NIHR Southampton Biomedical Research Centre [NIHR203319]) and the British Heart Foundation (RG/15/17/3174, SP/F/21/150013). For Open Access, the author has applied a Creative Commons Attribution (CC BY) licence to any Author Accepted Manuscript version arising from this submission. This work was supported by funding from a UK Research and Innovation Global Challenges Research Fund (GCRF) GROW Award (MR/P027938/1).

## CONFLICT OF INTEREST STATEMENT

KMG has received reimbursement for speaking at conferences sponsored by companies selling nutritional products and is part of an academic consortium that has received research funding from Abbott Nutrition, Nestec, BenevolentAI Bio Ltd. and Danone, outside the submitted work.

## ETHICS APPROVAL

The SWS was conducted in line with the guidelines provided in the Declaration of Helsinki and was approved by the Southampton and Southwest Hampshire Local Research Ethics Committee (08/H0502/95). Written informed consent was obtained from all participants.

## Supporting information


Tables S1–S9

## Data Availability

Data sharing not applicable – no new data generated.

## References

[1] Magee LA , Brown MA , Hall DR , Gupte S , Hennessy A , Karumanchi SA , et al. The 2021 International Society for the Study of Hypertension in Pregnancy classification, diagnosis & management recommendations for international practice. Pregnancy Hypertens. 2022;27:148–169. 35066406 10.1016/j.preghy.2021.09.008

[2] Malik R , Georgakis MK , Vujkovic M , Damrauer SM , Elliott P , Karhunen V , et al. Relationship between blood pressure and incident cardiovascular disease: linear and nonlinear Mendelian randomization analyses. Hypertension. 2021;77:2004–2013. 10.1161/HYPERTENSIONAHA.120.16534 33813844 PMC8115430

[3] Whelton PK , Carey RM , Aronow WS , Casey DE Jr , Collins KJ , Dennison Himmelfarb C , et al. 2017 ACC/AHA/AAPA/ABC/ACPM/AGS/APhA/ ASH/ASPC/NMA/PCNA guideline for the prevention, detection, evaluation, and management of high blood pressure in adults a report of the American College of Cardiology/American Heart Association Task Force on clinical practice guidelines. Hypertension. 2018;71:E13–E115. 29133356 10.1161/HYP.0000000000000065

[4] Slade LJ , Mistry HD , Bone JN , Wilson M , Blackman M , Syeda N , et al. American College of Cardiology/American Heart Association blood pressure categories – a systematic review of the relationship with adverse pregnancy outcomes. Am J Obstet Gynecol. 2023;228(4):418–429.e34. 36241079 10.1016/j.ajog.2022.10.004 PMC10239058

[5] Slade L , Wilson M , Mistry HD , Bone JN , Bello NA , Blackman M , et al. The 2017 American College of Cardiology/American Heart Association blood pressure categories in the second half of pregnancy – a systematic review of their association with adverse pregnancy outcomes. Am J Obstet Gynecol. 2023;229:101–117. 36657559 10.1016/j.ajog.2023.01.013

[6] Stevens SL , Wood S , Koshiaris C , Law K , Glasziou P , Stevens RJ , et al. Blood pressure variability and cardiovascular disease: systematic review and meta‐analysis. BMJ. 2016;354:i4098. 27511067 10.1136/bmj.i4098 PMC4979357

[7] Kim SA , Lee JD , Park JB . Differences in visit‐to‐visit blood pressure variability between normotensive and hypertensive pregnant women. Hypertens Res. 2019;42(1):67–74. 30315199 10.1038/s41440-018-0112-7

[8] Jieyu L , Yingying C , Tian G , Jiaxiang W , Jiawen L , Yingjie G , et al. Visit‐to‐visit blood pressure variability is associated with gestational hypertension and pre‐eclampsia. Pregnancy Hypertens. 2019;18:126–131. 31610398 10.1016/j.preghy.2019.09.009

[9] Magee LA , Singer J , Lee T , McManus RJ , Lay‐Flurrie S , Rey E , et al. Are blood pressure level and variability related to pregnancy outcome? Analysis of control of hypertension in pregnancy study data. Pregnancy Hypertens. 2020;19:87–93. 31927325 10.1016/j.preghy.2019.12.002

[10] Liu J , Yang L , Teng H , Cao Y , Wang J , Han B , et al. Visit‐to‐visit blood pressure variability and risk of adverse birth outcomes in pregnancies in East China. Hypertens Res. 2021;44(2):239–249. 32895496 10.1038/s41440-020-00544-7

[11] Gu Y , Shi H , Zeng W , Zheng Y , Yang M , Sun M , et al. Association between gestational visit‐to‐visit blood pressure variability and adverse neonatal outcomes. J Clin Hypertens. 2022;24:779–788. 10.1111/jch.14500 PMC9180330 35567772

[12] Bone JN , Magee LA , Singer J , Nathan H , Qureshi RN , Sacoor C , et al. Blood pressure thresholds in pregnancy for identifying maternal and infant risk: a secondary analysis of Community‐Level Interventions for Pre‐eclampsia (CLIP) trial data. Lancet Glob Health. 2021;9:e1119–e1128. 34237265 10.1016/S2214-109X(21)00219-9 PMC8295039

[13] Magee LA , Bone J , Owasil SB , Singer J , Lee T , Bellad MB , et al. Pregnancy outcomes and blood pressure visit‐to‐visit variability and level in three less‐developed countries. Hypertension. 2021;77:1714–1722. 33775120 10.1161/HYPERTENSIONAHA.120.16851 PMC8284372

[14] Inskip HM , Godfrey KM , Robinson SM , Law CM , Barker DJ , Cooper C , et al. Cohort profile: the Southampton Women's survey. Int J Epidemiol. 2006;35:42–48. 16195252 10.1093/ije/dyi202 PMC4579566

[15] National Institute for Health and Care Excellence NICE . Hypertension in pregnancy: diagnosis and management. NICE guidelines (NG133); 2019. p. 1–57. 31498578

[16] Whelton PK , Carey RM , Aronow WS , Casey DE Jr , Collins KJ , Dennison Himmelfarb C , et al. 2017 ACC/AHA/AAPA/ABC/ACPM/AGS/APhA/ASH/ASPC/NMA/PCNA guideline for the prevention, detection, evaluation, and management of high blood pressure in adults: executive summary: a report of the American college of cardiology/American Heart Association task force on clinical practice guidelines. Hypertension. 2018;71:1269–1324. 29133354 10.1161/HYP.0000000000000066

[17] Mancia G , Ferrari A , Gregorini L , Parati G , Pomidossi G , Bertinieri G , et al. Blood pressure and heart rate variabilities in normotensive and hypertensive human beings. Circ Res. 1983;53:96–104. 6861300 10.1161/01.res.53.1.96

[18] Papageorghiou AT , Kennedy SH , Salomon LJ , Altman DG , Ohuma EO , Stones W , et al. The INTERGROWTH‐21st fetal growth standards: toward the global integration of pregnancy and pediatric care. Am J Obstet Gynecol. 2018;218:S630–S640. 29422205 10.1016/j.ajog.2018.01.011

[19] Duffy JMN , Cairns AE , Richards‐Doran D , van ‘t Hooft J , Gale C , Brown M , et al. A core outcome set for pre‐eclampsia research: an international consensus development study. BJOG. 2020;127:1516–1526. 32416644 10.1111/1471-0528.16319

[20] Ranganathan P , Aggarwal R . Understanding the properties of diagnostic tests – part 2: likelihood ratios. Perspect Clin Res. 2018;9:99. 29862204 10.4103/picr.PICR_41_18 PMC5950618

[21] van Buuren S , Groothuis‐Oudshoorn CG . Mice: multivariate imputation by chained equations in R. J Stat Softw. 2011;45:8–16.

[22] Little RJA , Rubin DB . Statistical analysis with missing data. 2nd ed. Hoboken: John Wiley & Sons; 2002. 5.4.

[23] Magee LA , von Dadelszen P , Rey E , Ross S , Asztalos E , Murphy KE , et al. Less‐tight versus tight control of hypertension in pregnancy. N Engl J Med. 2015;372:407–417. 25629739 10.1056/NEJMoa1404595

[24] Tita AT , Szychowski JM , Boggess K , Dugoff L , Sibai B , Lawrence K , et al. Treatment for mild chronic hypertension during pregnancy. N Engl J Med. 2022;386:1781–1792. 35363951 10.1056/NEJMoa2201295 PMC9575330

[25] World Health Organisation . WHO recommendations for prevention and treatment of pre‐eclampsia and eclampsia. 2011 [accessed 2023 Nov 28]. Available from: https://www.who.int/publications/i/item/9789241548335 23741776

[26] Abalos E , Cuesta C , Carroli G , Qureshi Z , Widmer M , Vogel JP , et al. Pre‐eclampsia, eclampsia and adverse maternal and perinatal outcomes: a secondary analysis of the World Health Organization Multicountry Survey on Maternal and Newborn Health. BJOG. 2014;121:14–24. 10.1111/1471-0528.12629 24641531

[27] Magee LA , Nicolaides KH , von Dadelszen P . Preeclampsia. N Engl J Med. 2022;386:1817–1832. 35544388 10.1056/NEJMra2109523

[28] Nicolaides KH , Papastefanou I , Syngelaki A , Ashoor G , Akolekar R . Predictive performance for placental dysfunction related stillbirth of the competing risks model for small‐for‐gestational‐age fetuses. BJOG. 2022;129:1530–1537. 34919332 10.1111/1471-0528.17066

