# Supplementary material for: Blood pressure measurement and adverse pregnancy outcomes: A cohort study testing blood pressure variability and alternatives to 140/90 mmHg
Source: BJOG. 2023 Dec 6;131(7):1006–16. doi: 10.1111/1471-0528.17724 (PMC11256866; doi:10.1111/1471-0528.17724)
Supplement: Supplementary file 1 — Tables S1–S9 [file BJO-131-1006-s001.docx]

Supporting Information

Blood pressure measurement and adverse pregnancy outcomes – a cohort study testing blood pressure variability and alternatives to 140/90mmHg

Milly G Wilson ^1^

Jeffrey N Bone ^2,3^

Laura Slade ^4,5^

Hiten D Mistry ^1^

Joel Singer ^6^

Sarah R Crozier ^7,8^

Keith M Godfrey ^7,9^

Janis Baird ^7,8,9^

Peter von Dadelszen ^1*^

Laura A Magee ^1*^

** These authors contributed equally to the work.*

1. Department of Women and Children’s Health, School of Life Course and Population Sciences, Faculty of Medicine, King’s College London, London, UK;
2. British Columbia Children's Hospital Research Institute, University of British Columbia, Vancouver, Canada;
3. Department of Obstetrics and Gynaecology, University of British Columbia, Vancouver, Canada;
4. Robinson Research Institute, The University of Adelaide, South Australia, Australia; and
5. Department of Obstetrics and Gynaecology, Women’s and Children’s Hospital, Adelaide, Australia;
6. School of Population and Public Health, University of British Columbia, Vancouver, Canada;
7. MRC Lifecourse Epidemiology Centre, University of Southampton, Southampton, UK;
8. NIHR Applied Research Collaboration Wessex, Southampton Science Park, Southampton, UK;
9. NIHR Southampton Biomedical Research Centre, University of Southampton and University Hospital Southampton NHS Foundation Trust, UK.

**Address for correspondence:**

Professor Laura A. Magee

Addison House

Guy’s Campus

Great Maze Pond

London, UK

SE1 1UL

Laura.A.Magee@kcl.ac.uk

Tel: +44 (0)20 7848 9571

| Supplementary Material Index | | |
| --- | --- | --- |
| Table | Title | Page |
| S1 | Measures of BP Variability | 3 |
| S2 | BP Characteristics | 4 |
| S3 | BP Categorisation Before and After 20**^+0^**  Weeks’ Gestation, according to ACC/AHA Criteria | 5 |
| S4 | Systolic and Diastolic BP (%) Change From Booking | 6 |
| S5 | Correlation Coefficients Between Number of BP Measurements and BP Characteristics, in Main and Sensitivity Analyses | 7 |
| S6 | Sensitivity, Specificity, and Likelihood Ratios for ACC/AHA BP Categories and Pregnancy Outcomes (<20/≥20 Weeks’ Gestation) | 8 |
| S7 | Adjusted RRs for Measures of BP Variability and Pregnancy Outcomes | 10 |
| S8 | Sensitivity Analyses: Adjusted RRs For Relationship Between BPV and Pregnancy Outcomes, Excluding BP Values One-, Two-, Four-, and Six- Weeks from Delivery | 12 |
| S9 | Sensitivity Analyses: Adjusted RRs for Relationship Between BPV and Pregnancy Outcomes, Excluding and Exclusively Women with Chronic Hypertension | 16 |

| **Table S1.** Measures of BP Variability | |
| --- | --- |
| Measure | Formula |
| Inter-Individual Standard Deviation (SD) | 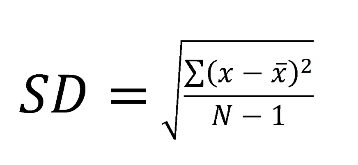 |
| Average Real Variability (ARV) | ***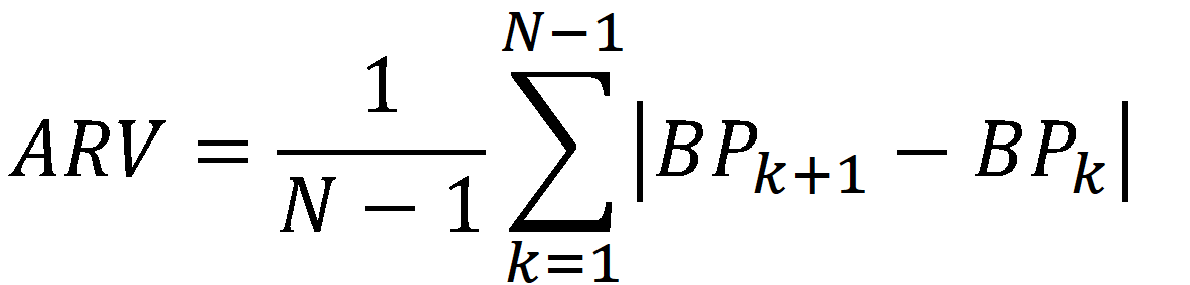Where,***  *N (number of valid BP measurements), k (order of BP measurements)* |
| Variability Independent of the Mean (VIM) | *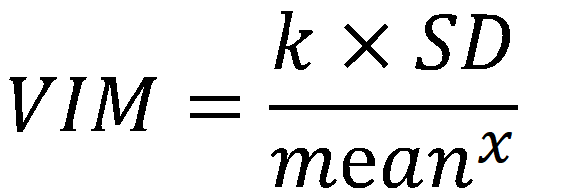*  ***Where****,*  *x is obtained from nonlinear regression analysis among the entire sample where SD = a*mean^x^.*  ***And,***  *k = mean(sample mean)^x^* |
| BP indicates blood pressure. | |

| **Table S2.** BP Characteristics | | |
| --- | --- | --- |
| **Variables,** (Median [Interquartile Range]) | **Systolic BP** | **Diastolic BP** |
| **Number of BP measurements during pregnancy** | 11.00 [9.00–13.00] | 11.00 [9.00–13.00] |
| <20^+0^ weeks’ gestation | 2.00 [2.00-3.00] | 2.00 [2.00-3.00] |
| **≥**20^+0^ weeks’ gestation | 9.00 [7.00-10.00] | 9.00 [7.00-10.00] |
| **BP level** (overall) | 112.0 [110.00–120.00] | 68.5 [60.00–70.00] |
| <20^+0^ weeks’ gestation | 110.0 [105.00–120.00] | 65.0 [60.00–70.00] |
| **≥**20^+0^ weeks’ gestation | 113.5 [110.00–120.00] | 69.5 [60.00–70.00] |
| **BPV** (overall) |  |  |
| *SD* | 8.17 [6.44–10.07] | 6.55 [5.22–8.02] |
| *ARV* | 7.50 [5.57–9.80] | 5.83 [4.33–7.56] |
| *VIM* | 8.23 [6.50-10.10] | 6.58 [5.28-8.07] |
| <20^+0^ weeks’ gestation |  |  |
| *SD* | 5.77 [2.12–8.56] | 4.62 [1.41–7.07] |
| *ARV* | 7.00 [2.00–10.00] | 5.00 [2.00–10.00] |
| *VIM* | 5.96 [2.17-8.70] | 4.46 [1.49-7.14] |
| **≥**20^+0^ weeks’ gestation |  |  |
| *SD* | 7.87 [6.02–9.90] | 6.40 [4.99–7.99] |
| *ARV* | 7.50 [5.33–10.00] | 5.80 [4.17–7.69] |
| *VIM* | 7.95 [6.10-9.93] | 6.40 [5.08-7.94] |
| ARV, average real variability; BP, blood pressure; BPV, blood pressure variability; SD, standard deviation; VIM, variability independent of the mean. | | |

| **Table S3.** BP Categorization Before and After 20**^+0^** Weeks’ Gestation, according to ACC/ AHA Criteria ǁ | | | | | |
| --- | --- | --- | --- | --- | --- |
|  | **At ≥20^+0^ weeks’ gestation** | | | | |
| **At <20^+0^ weeks’ gestation** | **‘Normal’ BP**  **(n=1150)** | **‘Elevated BP’**  **(n=813)** | **‘Stage 1 hypertension’**  **(n=770)** | **‘Non-severe stage 2 hypertension’ (n=252)** | **‘Severe stage 2 hypertension’ (n=18)** |
| **‘Normal’ BP** at <20^+0^ weeks (n=2122) | 1032 | 569* | 420**†** | 98 | 3 |
| **‘Elevated BP’** at <20^+0^ weeks (n=519) | 77* | 181* | 1968***†** | 62 | 3 |
| **‘Stage 1 hypertension’** at <20^+0^ weeks (n=263) | 12**†** | 44***†** | 129**†** | 67 | 11 |
| **‘Non-severe stage 2 hypertension’** at <20^+0^ weeks (n=41) | 4 | 3 | 12 | 21 | 1 |
| **‘Severe stage 2 hypertension’** at <20^+0^ weeks (n=1) | 0 | 0 | 1 | 0 | 0 |
| *No BP values at’ <20 weeks (n=57)* | 25 | 16 | 12 | 4 | 0 |
| ACC, American College of Cardiology; AHA, American Heart Association; BP, blood pressure; dBP, diastolic blood pressure; sBP, systolic blood pressure. Data are (N). Blood pressure is categorized as: ‘Normal BP’ (sBP <120 mm Hg and dBP <80 mm Hg), ‘Elevated BP’ (sBP 120–129 mm Hg and dBP <80 mm Hg), ‘Stage 1 hypertension’ (sBP 130–139 mm Hg or dBP 80–89 mm Hg, or both), ‘Non-severe stage 2 hypertension’ (sBP 140–159 mm Hg or dBP 90–109 mm Hg, or both), or ‘Severe stage 2 hypertension’ (sBP ≥160 mm Hg or dBP ≥110 mm Hg, or both).  ***** Women diagnosed with hypertension with the addition of the ‘Elevated BP’ category from the ACC/AHA criteria.  **†** Women diagnosed with hypertension with the addition of the ‘Stage 1 hypertension’ category from the ACC/AHA criteria.  ǁ Data shaded in **GREEN** describe women whose BP was similar or lower in the second half (as in the first half) of pregnancy. | | | | | |

| **Table S4.** Systolic and Diastolic BP (%) Change From Booking | | | | | | |
| --- | --- | --- | --- | --- | --- | --- |
|  | Systolic BP (median [IQR]) | | | Diastolic BP (median [IQR]) | | |
| Outcome | BP at booking | Peak BP ≥20 weeks’ | % change from booking BP | BP at booking | Peak BP ≥20 weeks’ | % change from booking BP |
| Overall | 110.0 [105.0-120.0] | 125.0 [120.0-135.0] | 12.5 [5.7-20.8] | 68.0 [60.0-70.0] | 79.0 [70.0-82.0] | 16.7 [7.1-28.6] |
| Gestational hypertension | | | | | | |
| *Yes* | 112.0 [110.0-120.0] | 146.5 [140.0-155.0] | 30.0 [20.0-40.9] | 70.0 [60.0-75.0] | 95.0 [90.0-100.6] | 40.0 [25.2-50.8] |
| *No* | 110.0 [105.0-120.0] | 125.0 [120.0-132.0] | 11.1 [5.0-20.0] | 66.0 [60.0-70.0] | 78.0 [70.0-80.0] | 16.7 [6.7-26.7] |
| Severe hypertension | | | | | | |
| *Yes* | 120.0 [115.0-140.0] | 163.0 [160.0-170.0] | 34.4 [19.1-46.8] | 75.0 [70.0-80.0] | 102.8 [95.3-109.9] | 35.0 [22.5-54.8] |
| *No* | 110.0 [105.0-120.0] | 125.0 [120.0-134.0] | 11.7 [5.3-20.0] | 66.0 [60.0-70.0] | 78.0 [70.0-81.0] | 16.7 [7.1-27.3] |
| Preeclampsia | | | | | | |
| *Yes* | 115.0 [106.2-124.5] | 151.6 [142.0-167.0] | 34.8 [21.0-46.0] | 70.0 [60.0-77.5] | 100.0 [91.7-105.1] | 43.6 [29.7-57.0] |
| *No* | 110.0 [105.0-120.0] | 125.0 [120.0-134.0] | 11.7 [5.2-20.0] | 67.0 [60.0-70.0] | 78.0 [70.0-81.0] | 16.7 [7.1-27.3] |
| PTB <37 | | | | | | |
| *Yes* | 110.0 [109.5-120.0] | 128.0 [120.0-140.0] | 10.0 [2.3-23.0] | 70.0 [60.0-74.5] | 78.0 [70.0-86.3] | 16.7 [2.7-30.0] |
| *No* | 110.0 [105.0-120.0] | 125.0 [120.0-135.0] | 12.5 [6.1-20.8] | 68.0 [60.0-70.0] | 79.0 [70.0-82.0] | 16.7 [7.1-28.6] |
| SGA | | | | | | |
| *Yes* | 110.0 [100.0-120.0] | 126.0 [120.0-140.0] | 16.7 [7.1-25.0] | 65.0 [60.0-70.0] | 80.0 [70.0-90.0] | 21.4 [7.7-33.3] |
| *No* | 110.0 [105.0-120.0] | 125.0 [120.0-135.0] | 12.0 [5.6-20.4] | 68.0 [60.0-70.0] | 78.0 [70.0-82.0] | 16.7 [7.1-28.3] |
| NICU admission | | | | | | |
| *Yes* | 110.0 [105.0-120.0] | 130.0 [120.0-140.0] | 14.9 [6.8-25.0] | 70.0 [60.0-75.0] | 80.0 [70.0-85.0] | 16.7 [6.6-25.4] |
| *No* | 110.0 [105.0-120.0] | 125.0 [120.0-1350] | 12.2 [5.6-20.4] | 67.0 [60.0-70.0] | 78.0 [70.0-82.0] | 16.7 [7.1-28.6] |
| BP indicates blood pressure; NICU, neonatal intensive care unit admission; PTB, preterm birth; SGA, small-for-gestational-age. | | | | | | |

| **Table S5.** Correlation Coefficients Between Number of BP Measurements and BP Characteristics, in Main and Sensitivity Analyses | | | | | |
| --- | --- | --- | --- | --- | --- |
| **BP Characteristic** | **Overall (N=3003)** | **One-Week Analysis (N=3003)** | **Two-Week Analysis (N=3003)** | **Four-Week Analysis (N=3002)** | **Six-Week Analysis (N=3001)** |
| **Mean** |  | | | | |
| *Systolic* | 0.29 | 0.26 | 0.24 | 0.21 | 0.18 |
| *Diastolic* | 0.28 | 0.25 | 0.23 | 0.20 | 0.16 |
| **ARV** |  | | | | |
| *Systolic* | 0.01 | 0.01 | 0.01 | -0.02 | -0.06 |
| *Diastolic* | 0.04 | 0.05 | 0.05 | 0.04 | 0.04 |
| **SD** |  | | | | |
| *Systolic* | 0.15 | 0.14 | 0.12 | 0.08 | 0.07 |
| *Diastolic* | 0.17 | 0.19 | 0.16 | 0.14 | 0.13 |
| **VIM** |  | | | | |
| *Systolic* | 0.06 | 0.02 | 0.06 | 0.04 | 0.04 |
| *Diastolic* | 0.10 | 0.07 | 0.12 | 0.11 | 0.11 |
| ARV indicates average real variability; BP, blood pressure; dBP, diastolic blood pressure; sBP, systolic blood pressure; SD; standard deviation; VIM, variability independent of the mean.  Pearson’s product-moment correlation coefficients with associated P values. P ≤0.05 is considered statistically significant.  Interpretation: coefficient between ± 0.50 and ± 1 = strong correlation; between ± 0.30 and ± 0.49 = moderate correlation; below ± 0. 29 = small correlation.  One-, two-, four- and six-week analyses are mean, SD, ARV, and VIM (for both sBP and dBP) calculated excluding BP values 7, 14, 28 and 42 days from delivery respectively. | | | | | |

| **Table S6.** Sensitivity and Specificity of ACC/AHA BP Categories and Pregnancy Outcomes (<20/≥20 Weeks’ Gestation)***** | | | |
| --- | --- | --- | --- |
| **<20 Weeks’ Gestation** | | | |
|  | **Events, n (%)†** | **Sensitivity (95% CI)** | **Specificity LR (95% CI)** |
| **Pre-eclampsia** | | | |
| ‘Normal BP’ | 50 (2.35) | *Ref* | *Ref* |
| ‘Elevated BP’ | 14 (2.69) | 0.43 (0.33, 0.54) | 0.73 (0.71, 0.74) |
| ‘Stage 1 Hypertension’ | 19 (7.22) | 0.27 (0.18, 0.38) | 0.90 (0.89, 0.91) |
| ‘Non-Severe Stage 2 Hypertension’ | 5 (12.2) | 0.06 (0.02, 0.13) | 0.99 (0.98, 0.99) |
| ‘Severe Stage 2 Hypertension’ | 0 (0) | 0.00 (0.00, 0.04) | 1.00 (1.00, 1.00) |
| **PTB <37 weeks** | | | |
| ‘Normal BP’ | 117 (5.50) | *Ref* | *Ref* |
| ‘Elevated BP’ | 20 (3.85) | 0.29 (0.22, 0.37) | 0.72 (0.70, 0.74) |
| ‘Stage 1 Hypertension’ | 23 (8.75) | 0.17 (0.12, 0.24) | 0.90 (0.89, 0.91) |
| ‘Non-Severe Stage 2 Hypertension’ | 5 (12.2) | 0.03 (0.01, 0.07) | 0.99 (0.98, 0.99) |
| ‘Severe Stage 2 Hypertension’ | 0 (0) | 0.00 (0.00, 0.02) | 1.00 (1.00, 1.00) |
| **SGA** | | | |
| ‘Normal BP’ | 116 (5.46) | *Ref* | *Ref* |
| ‘Elevated BP’ | 23 (4.42) | 0.31 (0.24, 0.38) | 0.72 (0.70, 0.74) |
| ‘Stage 1 Hypertension’ | 25 (9.51) | 0.17 (0.11, 0.23) | 0.90 (0.89, 0.91) |
| ‘Non-Severe Stage 2 Hypertension’ | 3 (7.32) | 0.02 (0.00, 0.05) | 0.99 (0.98, 0.99) |
| ‘Severe Stage 2 Hypertension’ | 0 (0) | 0.00 (0.00, 0.02) | 1.00 (1.00, 1.00) |
| **NICU Admission** | | | |
| ‘Normal BP’ | 121 (5.69) | *Ref* | *Ref* |
| ‘Elevated BP’ | 32 (6.15) | 0.33 (0.26, 0.41) | 0.72 (0.71, 0.74) |
| ‘Stage 1 Hypertension’ | 23 (8.75) | 0.15 (0.11, 0.22) | 0.90 (0.89, 0.91) |
| ‘Non-Severe Stage 2 Hypertension’ | 5 (12.2) | 0.03 (0.01, 0.06) | 0.99 (0.98, 0.99) |
| ‘Severe Stage 2 Hypertension’ | 0 (0) | 0.00 (0.00, 0.02) | 1.00 (1.00, 1.00) |
| **≥20 Weeks’ Gestation** | | | |
|  | **Events, n (%)†** | **Sensitivity (95% CI)** | **Specificity LR (95% CI)** |
| **Pre-eclampsia** | | | |
| ‘Normal BP’ | 4 (0.35) | *Ref* | *Ref* |
| ‘Elevated BP’ | 5 (0.62) | 0.95 (0.89-0.99) | 0.39 (0.38-0.41) |
| ‘Stage 1 Hypertension’ | 22 (2.86) | 0.90 (0.81-0.95) | 0.67 (0.65-0.69) |
| ‘Non-Severe Stage 2 Hypertension’ | 46 (18.25) | 0.65 (0.54-0.75) | 0.93 (0.92-0.94) |
| ‘Severe Stage 2 Hypertension’ | 11 (61.1) | 0.12 (0.06-0.21) | 1.00 (1.00-1.00) |
| **PTB <37 weeks** | | | |
| ‘Normal BP’ | 78 (6.78) | *Ref* | *Ref* |
| ‘Elevated BP’ | 25 (3.08) | 0.53 (0.45-0.61) | 0.38 (0.36-0.40) |
| ‘Stage 1 Hypertension’ | 39 (5.06) | 0.38 (0.31-0.46) | 0.66 (0.64-0.67) |
| ‘Non-Severe Stage 2 Hypertension’ | 20 (7.94) | 0.15 (0.10-0.21) | 0.91 (0.90-0.92) |
| ‘Severe Stage 2 Hypertension’ | 5 (27.7) | 0.03 (0.01-0.07) | 1.00 (0.99-1.00) |
| **SGA** | | | |
| ‘Normal BP’ | 67 (5.83) | *Ref* | *Ref* |
| ‘Elevated BP’ | 37 (4.55) | 0.60 (0.53-0.68) | 0.38 (0.36-0.40) |
| ‘Stage 1 Hypertension’ | 34 (4.42) | 0.38 (0.31-0.46) | 0.66 (0.64-0.67) |
| ‘Non-Severe Stage 2 Hypertension’ | 26 (10.32) | 0.18 (0.13-0.25) | 0.92 (0.90-0.93) |
| ‘Severe Stage 2 Hypertension’ | 5 (27.7) | 0.03 (0.01-0.07) | 1.00 (0.99-1.00) |
| **NICU Admission** | | | |
| ‘Normal BP’ | 70 (6.09) | *Ref* | *Ref* |
| ‘Elevated BP’ | 32 (3.94) | 0.62 (0.54-0.69) | 0.38 (0.36-0.40) |
| ‘Stage 1 Hypertension’ | 53 (6.88) | 0.44 (0.37-0.52) | 0.66 (0.64-0.68) |
| ‘Non-Severe Stage 2 Hypertension’ | 23 (9.13) | 0.15 (0.10-0.21) | 0.91 (0.90-0.92) |
| ‘Severe Stage 2 Hypertension’ | 5 (27.7) | 0.03 (0.01-0.06) | 1.00 (0.99-1.00) |
| ACC indicates American College of Cardiology; AHA, American Heart Association; BP, blood pressure; CI, confidence interval; LR, likelihood ratio; NICU, neonatal intensive care unit; PTB, preterm birth; SGA, small-for-gestational-age.  ***** BP is categorized as: ‘Normal BP’ (sBP <120 mm Hg and dBP <80 mm Hg), ‘Elevated BP’ (sBP 120–129 mm Hg and dBP <80 mm Hg), ‘Stage 1 hypertension’ (sBP 130–139 mm Hg or dBP 80–89 mm Hg, or both), and ‘Stage 2 hypertension’ (sBP ≥140 mm Hg or dBP ≥90 mm Hg, or both), including non-severe ‘Stage 2 hypertension’ (sBP 140–159 mm Hg or dBP 90–109 mm Hg, or both) and severe ‘Stage 2 hypertension’ (sBP ≥160 mm Hg or dBP ≥110 mm Hg, or both). All analyses were adjusted for maternal age, body mass index, parity, and smoking status. A positive LR **≥**5.00 or a negative LR <0.20 was considered good.  **†** Events only include women in the category specified; the denominator is women with complete outcome data. | | | |

| **Table S7.** Adjusted RRs for Measures of BP Variability and Pregnancy Outcomes | | | |
| --- | --- | --- | --- |
| **Outcomes** | | **Systolic BP** | **Diastolic BP** |
| **Gestational Hypertension** | |  |  |
| *SD* | *Adjusted* | 1.15 (1.11–1.19) | 1.27 (1.22–1.33) |
|  | *Crude* | 1.22 (1.17–1.26) | 1.38 (1.33–1.44) |
| *ARV* | *Adjusted* | 1.06 (1.02–1.10) | 1.09 (1.06–1.13) |
|  | *Crude* | 1.12 (1.10–1.15) | 1.14 (1.12–1.16) |
| *VIM* | *Adjusted* | 1.18 (1.13–1.24) | 1.33 (1.27–1.39) |
|  | *Crude* | 1.24 (1.20–1.28) | 1.33 (1.29–1.38) |
| **Severe Hypertension** | |  |  |
| *SD* | *Adjusted* | 1.16 (1.11–1.21) | 1.28 (1.20–1.37) |
|  | *Crude* | 1.26 (1.20–1.31) | 1.42 (1.36–1.48) |
| *ARV* | *Adjusted* | 1.07 (1.02–1.12) | 1.15 (1.10–1.20) |
|  | *Crude* | 1.19 (1.15–1.22) | 1.18 (1.14–1.22) |
| *VIM* | *Adjusted* | 1.21 (1.15–1.27) | 1.35 (1.26–1.45) |
|  | *Crude* | 1.28 (1.23–1.33) | 1.34 (1.28–1.40) |
| **Pre-eclampsia** | |  |  |
| *SD* | *Adjusted* | 1.16 (1.11–1.21) | 1.32 (1.25–1.40) |
|  | *Crude* | 1.24 (1.19–1.30) | 1.45 (1.38–1.52) |
| *ARV* | *Adjusted* | 1.07 (1.03–1.12) | 1.13 (1.09–1.18) |
|  | *Crude* | 1.16 (1.13–1.19) | 1.18 (1.15–1.21) |
| *VIM* | *Adjusted* | 1.20 (1.14–1.26) | 1.39 (1.32–1.47) |
|  | *Crude* | 1.28 (1.23–1.34) | 1.40 (1.34–1.46) |
| **PTB <37** | |  |  |
| *SD* | *Adjusted* | 1.09 (1.05–1.14) | 1.10 (1.03–1.17) |
|  | *Crude* | 1.11 (1.00–1.15) | 1.12 (1.06–1.19) |
| *ARV* | *Adjusted* | 1.07 (1.04–1.10) | 1.08 (1.04–1.12) |
|  | *Crude* | 1.08 (1.05–1.12) | 1.09 (1.05–1.14) |
| *VIM* | *Adjusted* | 1.09 (1.05–1.14) | 1.10 (1.03–1.17) |
|  | *Crude* | 1.10 (1.05–1.16) | 1.10 (1.03–1.18) |
| **SGA** | |  |  |
| *SD* | *Adjusted* | 1.07 (1.03–1.11) | 1.05 (0.99–1.11) |
|  | *Crude* | 1.10 (1.06–1.14) | 1.11 (1.04–1.17) |
| *ARV* | *Adjusted* | 1.02 (0.99–1.05) | 1.02 (0.98–1.06) |
|  | *Crude* | 1.04 (1.00–1.08) | 1.05 (1.00–1.10) |
| *VIM* | *Adjusted* | 1.07 (1.03–1.12) | 1.05 (0.99–1.11) |
|  | *Crude* | 1.10 (1.05–1.15) | 1.08 (1.01–1.15) |
| **NICU Admission** | |  |  |
| *SD* | *Adjusted* | 1.09 (1.05–1.13) | 1.03 (0.96–1.10) |
|  | *Crude* | 1.10 (1.06–1.14) | 1.06 (0.99–1.13) |
| *ARV* | *Adjusted* | 1.05 (1.02–1.09) | 1.03 (0.98–1.09) |
|  | *Crude* | 1.06 (1.03–1.10) | 1.05 (0.99–1.10) |
| *VIM* | *Adjusted* | 1.09 (1.05–1.14) | 1.02 (0.95–1.10) |
|  | *Crude* | 1.10 (1.05–1.14) | 1.03 (0.96–1.10) |
| ARV indicates average real variability; BP, blood pressure; NICU, neonatal intensive care unit; PTB, preterm birth; SD; standard deviation; RR, relative risk; SGA, small-for-gestational-age; VIM, variability independent of the mean.  Data are aRR (95% CI) (adjusted risk ratio (95% confidence interval). Models are adjusted for maternal age, BMI, mean sBP/dBP and smoking status. | | | |

| **Table S8.** Sensitivity Analyses: Adjusted RRs For Relationship Between BPV and Pregnancy Outcomes, Excluding BP Values One-, Two-, Four-, and Six- Weeks from Delivery | | | | | | | |
| --- | --- | --- | --- | --- | --- | --- | --- |
| **One- Week Sensitivity Analysis** | | | | **Two- Week Sensitivity Analysis** | | | |
| Outcomes | | Systolic BP | Diastolic BP | Outcomes | | Systolic BP | Diastolic BP |
| **Gestational Hypertension** | |  |  | **Gestational Hypertension** | |  |  |
| *SD* | *Adjusted* | 1.18 (1.13–1.23) | 1.23 (1.17–1.30) | *SD* | *Adjusted* | 1.14 (1.09–1.19) | 1.20 (1.14–1.26) |
|  | *Crude* | 1.26 (1.22–1.31) | 1.36 (1.29–1.43) |  | *Crude* | 1.21 (1.17–1.26) | 1.28 (1.22–1.35) |
| *ARV* | *Adjusted* | 1.03 (0.99–1.08) | 1.06 (1.01–1.12) | *ARV* | *Adjusted* | 1.02 (0.98–1.07) | 1.07 (1.01–1.12) |
|  | *Crude* | 1.09 (1.05–1.13) | 1.13 (1.08–1.18) |  | *Crude* | 1.07 (1.03–1.11) | 1.11 (1.06–1.16) |
| *VIM* | *Adjusted* | 1.20 (1.15–1.26) | 1.27 (1.21–1.34) | *VIM* | *Adjusted* | 1.16 (1.10–1.21) | 1.22 (1.16–1.29) |
|  | *Crude* | 1.23 (1.18–1.28) | 1.29 (1.23–1.36) |  | *Crude* | 1.17 (1.12–1.23) | 1.23 (1.16–1.30) |
| **Severe Hypertension** | |  |  | **Severe Hypertension** | |  |  |
| *SD* | *Adjusted* | 1.20 (1.14–1.26) | 1.25 (1.16–1.34) | *SD* | *Adjusted* | 1.18 (1.11–1.24) | 1.27 (1.18–1.36) |
|  | *Crude* | 1.38 (1.32–1.44) | 1.43 (1.35–1.52) |  | *Crude* | 1.37 (1.32–1.42) | 1.38 (1.29–1.47) |
| *ARV* | *Adjusted* | 1.06 (1.00–1.12) | 1.19 (1.10–1.28) | *ARV* | *Adjusted* | 1.05 (1.00–1.10) | 1.18 (1.10–1.27) |
|  | *Crude* | 1.21 (1.17–1.25) | 1.27 (1.20–1.34) |  | *Crude* | 1.19 (1.15–1.23) | 1.23 (1.17–1.29) |
| *VIM* | *Adjusted* | 1.25 (1.19–1.32) | 1.30 (1.20–1.40) | *VIM* | *Adjusted* | 1.22 (1.15–1.29) | 1.30 (1.22–1.40) |
|  | *Crude* | 1.32 (1.26–1.37) | 1.32 (1.24–1.41) |  | *Crude* | 1.30 (1.24–1.36) | 1.30 (1.21–1.39) |
| **Preeclampsia** | |  |  | **Preeclampsia** | |  |  |
| *SD* | *Adjusted* | 1.20 (1.14–1.26) | 1.28 (1.19–1.37) | *SD* | *Adjusted* | 1.16 (1.10–1.23) | 1.27 (1.19–1.36) |
|  | *Crude* | 1.32 (1.26–1.37) | 1.43 (1.34–1.51) |  | *Crude* | 1.28 (1.22–1.34) | 1.37 (1.28–1.46) |
| *ARV* | *Adjusted* | 1.05 (1.00–1.10) | 1.14 (1.08–1.21) | *ARV* | *Adjusted* | 1.04 (1.00–1.09) | 1.15 (1.09–1.21) |
|  | *Crude* | 1.14 (1.09–1.19) | 1.22 (1.16–1.29) |  | *Crude* | 1.11 (1.06–1.17) | 1.20 (1.14–1.26) |
| *VIM* | *Adjusted* | 1.23 (1.16–1.29) | 1.33 (1.24–1.43) | *VIM* | *Adjusted* | 1.19 (1.12–1.26) | 1.31 (1.23–1.40) |
|  | *Crude* | 1.28 (1.21–1.34) | 1.36 (1.27–1.45) |  | *Crude* | 1.23 (1.16–1.31) | 1.32 (1.23–1.41) |
| **PTB <37** | |  |  | **PTB <37** | |  |  |
| *SD* | *Adjusted* | 1.06 (1.01–1.12) | 1.06 (0.98–1.14) | *SD* | *Adjusted* | 1.05 (1.00–1.11) | 1.06 (0.99–1.14) |
|  | *Crude* | 1.08 (1.02–1.14) | 1.08 (1.00–1.16) |  | *Crude* | 1.07 (1.01–1.13) | 1.08 (1.00–1.16) |
| *ARV* | *Adjusted* | 1.04 (1.00–1.09) | 1.05 (0.99–1.12) | *ARV* | *Adjusted* | 1.03 (0.99–1.07) | 1.05 (0.99–1.11) |
|  | *Crude* | 1.05 (1.01–1.10) | 1.06 (0.99–1.13) |  | *Crude* | 1.04 (1.00–1.09) | 1.06 (1.00–1.12) |
| *VIM* | *Adjusted* | 1.06 (1.00–1.12) | 1.05 (0.98–1.14) | *VIM* | *Adjusted* | 1.05 (0.99–1.11) | 1.06 (0.98–1.14) |
|  | *Crude* | 1.06 (1.00–1.13) | 1.06 (0.98–1.15) |  | *Crude* | 1.05 (0.99–1.12) | 1.06 (0.99–1.15) |
| **SGA** | |  |  | **SGA** | |  |  |
| *SD* | *Adjusted* | 1.06 (1.01–1.11) | 1.02 (0.96–1.09) | *SD* | *Adjusted* | 1.06 (1.01–1.12) | 1.02 (0.96–1.10) |
|  | *Crude* | 1.08 (1.03–1.14) | 1.07 (0.99–1.14) |  | *Crude* | 1.08 (1.03–1.14) | 1.06 (0.98–1.14) |
| *ARV* | *Adjusted* | 1.00 (0.96–1.04) | 1.01 (0.96–1.08) | *ARV* | *Adjusted* | 1.00 (0.96–1.04) | 1.01 (0.95–1.07) |
|  | *Crude* | 1.01 (0.97–1.05) | 1.03 (0.97–1.1) |  | *Crude* | 1.01 (0.97–1.06) | 1.02 (0.96–1.09) |
| *VIM* | *Adjusted* | 1.06 (1.01–1.11) | 1.02 (0.95–1.09) | *VIM* | *Adjusted* | 1.06 (1.01–1.12) | 1.02 (0.95–1.09) |
|  | *Crude* | 1.08 (1.02–1.14) | 1.04 (0.97–1.12) |  | *Crude* | 1.08 (1.03–1.14) | 1.04 (0.96–1.12) |
| **NICU Admission** | |  |  | **NICU Admission** | |  |  |
| *SD* | *Adjusted* | 1.07 (1.02–1.12) | 0.99 (0.92–1.07) | *SD* | *Adjusted* | 1.06 (1.02–1.12) | 1.01 (0.94–1.08) |
|  | *Crude* | 1.09 (1.04–1.14) | 1.02 (0.95–1.10) |  | *Crude* | 1.08 (1.03–1.13) | 1.03 (0.96–1.11) |
| *ARV* | *Adjusted* | 1.03 (0.99–1.07) | 1.00 (0.94–1.07) | *ARV* | *Adjusted* | 1.03 (0.99–1.07) | 1.01 (0.95–1.07) |
|  | *Crude* | 1.04 (1.00–1.08) | 1.02 (0.96–1.08) |  | *Crude* | 1.03 (0.99–1.08) | 1.02 (0.97–1.08) |
| *VIM* | *Adjusted* | 1.07 (1.02–1.12) | 0.99 (0.92–1.07) | *VIM* | *Adjusted* | 1.06 (1.01–1.12) | 1.01 (0.94–1.09) |
|  | *Crude* | 1.07 (1.02–1.13) | 1.00 (0.92–1.07) |  | *Crude* | 1.06 (1.01–1.12) | 1.02 (0.95–1.09) |
| **Four- Week Sensitivity Analysis** | | | | **Six- Week Sensitivity Analysis** | | | |
| Outcomes | | Systolic BP | Diastolic BP | Outcomes | | Systolic BP | Diastolic BP |
| **Gestational Hypertension** | |  |  | **Gestational Hypertension** | |  |  |
| *SD* | *Adjusted* | 1.08 (1.03–1.13) | 1.12 (1.06–1.18) | *SD* | *Adjusted* | 1.04 (0.99–1.09) | 1.06 (1.00–1.12) |
|  | *Crude* | 1.13 (1.08–1.19) | 1.16 (1.10–1.22) |  | *Crude* | 1.07 (1.02–1.13) | 1.08 (1.02–1.14) |
| *ARV* | *Adjusted* | 1.02 (0.98–1.06) | 1.05 (1.00–1.10) | *ARV* | *Adjusted* | 1.00 (0.97–1.04) | 1.03 (0.98–1.08) |
|  | *Crude* | 1.05 (1.01–1.08) | 1.07 (1.03–1.12) |  | *Crude* | 1.02 (0.98–1.06) | 1.05 (1.00–1.09) |
| *VIM* | *Adjusted* | 1.09 (1.04–1.14) | 1.13 (1.06–1.19) | *VIM* | *Adjusted* | 1.04 (0.99–1.10) | 1.06 (1.00–1.12) |
|  | *Crude* | 1.10 (1.05–1.16) | 1.12 (1.06–1.18) |  | *Crude* | 1.04 (0.99–1.10) | 1.04 (0.99–1.10) |
| **Severe Hypertension** | |  |  | **Severe Hypertension** | |  |  |
| *SD* | *Adjusted* | 1.13 (1.08–1.19) | 1.26 (1.19–1.34) | *SD* | *Adjusted* | 1.08 (1.03–1.14) | 1.16 (1.08–1.25) |
|  | *Crude* | 1.29 (1.24–1.33) | 1.29 (1.21–1.38) |  | *Crude* | 1.19 (1.13–1.26) | 1.19 (1.11–1.26) |
| *ARV* | *Adjusted* | 1.05 (1.01–1.09) | 1.15 (1.08–1.23) | *ARV* | *Adjusted* | 1.03 (1.00–1.07) | 1.09 (1.02–1.16) |
|  | *Crude* | 1.15 (1.11–1.18) | 1.18 (1.12–1.24) |  | *Crude* | 1.10 (1.06–1.14) | 1.10 (1.04–1.16) |
| *VIM* | *Adjusted* | 1.16 (1.10–1.22) | 1.27 (1.20–1.35) | *VIM* | *Adjusted* | 1.09 (1.03–1.15) | 1.17 (1.09–1.27) |
|  | *Crude* | 1.23 (1.17–1.29) | 1.23 (1.16–1.31) |  | *Crude* | 1.13 (1.06–1.21) | 1.13 (1.06–1.21) |
| **Preeclampsia** | |  |  | **Preeclampsia** | |  |  |
| *SD* | *Adjusted* | 1.09 (1.04–1.15) | 1.23 (1.17–1.30) | *SD* | *Adjusted* | 1.04 (0.97–1.10) | 1.13 (1.06–1.22) |
|  | *Crude* | 1.17 (1.11–1.24) | 1.26 (1.19–1.35) |  | *Crude* | 1.08 (1.00–1.16) | 1.15 (1.07–1.23) |
| *ARV* | *Adjusted* | 1.04 (0.99–1.08) | 1.14 (1.08–1.20) | *ARV* | *Adjusted* | 1.02 (0.97–1.07) | 1.09 (1.03–1.15) |
|  | *Crude* | 1.08 (1.03–1.13) | 1.16 (1.10–1.22) |  | *Crude* | 1.04 (0.99–1.1) | 1.10 (1.04–1.15) |
| *VIM* | *Adjusted* | 1.10 (1.04–1.17) | 1.25 (1.18–1.32) | *VIM* | *Adjusted* | 1.04 (0.97–1.11) | 1.14 (1.05–1.22) |
|  | *Crude* | 1.13 (1.06–1.21) | 1.23 (1.15–1.31) |  | *Crude* | 1.04 (0.97–1.13) | 1.11 (1.04–1.19) |
| **PTB <37** | |  |  | **PTB <37** | |  |  |
| *SD* | *Adjusted* | 1.03 (0.98–1.08) | 1.04 (0.98–1.11) | *SD* | *Adjusted* | 1.00 (0.95–1.06) | 1.03 (0.96–1.09) |
|  | *Crude* | 1.04 (0.98–1.1) | 1.06 (0.99–1.13) |  | *Crude* | 1.01 (0.95–1.07) | 1.04 (0.97–1.11) |
| *ARV* | *Adjusted* | 1.02 (0.98–1.06) | 1.02 (0.97–1.08) | *ARV* | *Adjusted* | 1.00 (0.96–1.04) | 1.02 (0.98–1.08) |
|  | *Crude* | 1.02 (0.98–1.07) | 1.03 (0.98–1.08) |  | *Crude* | 1.00 (0.96–1.04) | 1.03 (0.98–1.08) |
| *VIM* | *Adjusted* | 1.02 (0.97–1.08) | 1.04 (0.98–1.11) | *VIM* | *Adjusted* | 1.00 (0.94–1.06) | 1.03 (0.96–1.09) |
|  | *Crude* | 1.03 (0.97–1.09) | 1.05 (0.98–1.12) |  | *Crude* | 1.00 (0.94–1.06) | 1.03 (0.96–1.10) |
| **SGA** | |  |  | **SGA** | |  |  |
| *SD* | *Adjusted* | 1.05 (1.00–1.09) | 1.01 (0.95–1.08) | *SD* | *Adjusted* | 1.00 (0.95–1.05) | 0.97 (0.91–1.03) |
|  | *Crude* | 1.06 (1.01–1.11) | 1.03 (0.96–1.11) |  | *Crude* | 1.00 (0.95–1.05) | 0.97 (0.91–1.04) |
| *ARV* | *Adjusted* | 0.99 (0.95–1.03) | 1.00 (0.94–1.05) | *ARV* | *Adjusted* | 0.97 (0.94–1.01) | 0.98 (0.93–1.03) |
|  | *Crude* | 1.00 (0.96–1.04) | 1.01 (0.95–1.06) |  | *Crude* | 0.98 (0.94–1.01) | 0.98 (0.93–1.04) |
| *VIM* | *Adjusted* | 1.05 (1.00–1.10) | 1.01 (0.94–1.08) | *VIM* | *Adjusted* | 1.00 (0.95–1.05) | 0.96 (0.90–1.03) |
|  | *Crude* | 1.06 (1.01–1.11) | 1.02 (0.95–1.09) |  | *Crude* | 1.00 (0.95–1.05) | 0.97 (0.90–1.03) |
| **NICU Admission** | |  |  | **NICU Admission** | |  |  |
| *SD* | *Adjusted* | 1.03 (0.99–1.08) | 1.00 (0.94–1.06) | *SD* | *Adjusted* | 1.01 (0.96–1.06) | 1.00 (0.94–1.06) |
|  | *Crude* | 1.04 (0.99–1.09) | 1.01 (0.95–1.08) |  | *Crude* | 1.02 (0.97–1.07) | 1.01 (0.95–1.07) |
| *ARV* | *Adjusted* | 1.00 (0.97–1.04) | 0.99 (0.94–1.04) | *ARV* | *Adjusted* | 0.98 (0.95–1.02) | 0.99 (0.95–1.04) |
|  | *Crude* | 1.01 (0.97–1.05) | 1.00 (0.95–1.05) |  | *Crude* | 0.99 (0.95–1.03) | 1.00 (0.96–1.05) |
| *VIM* | *Adjusted* | 1.03 (0.99–1.08) | 1.00 (0.94–1.07) | *VIM* | *Adjusted* | 1.01 (0.96–1.06) | 1.00 (0.94–1.06) |
|  | *Crude* | 1.03 (0.98–1.08) | 1.00 (0.94–1.07) |  | *Crude* | 1.01 (0.96–1.06) | 1.00 (0.94–1.06) |
| ARV indicates average real variability; BP, blood pressure; dBP, diastolic blood pressure; NICU, neonatal intensive care unit; PTB, preterm birth; sBP, systolic blood pressure; SD; standard deviation; SGA, small-for-gestational-age; RR, risk ratio; VIM, variability independent of the mean.  Data are aRR (95% CI) (adjusted relative risk (95% confidence interval). Models are adjusted for maternal age, BMI, mean sBP/dBP and smoking status.  One-, two-, four- and six-week analyses are SD and ARV (for both sBP and dBP) calculated excluding BP values 7, 14, 28 and 42 days from delivery respectively. | | | | | | | |

| **Table S9.**  Sensitivity Analyses: Adjusted RRs for Relationship Between BPV and Pregnancy Outcomes, Excluding and Exclusively Women with Chronic Hypertension | | | | | | | |
| --- | --- | --- | --- | --- | --- | --- | --- |
| **Excluding 213 Women with Chronic Hypertension (N=2790)** | | | | **Restricting only to Women with Chronic Hypertension (N=213)** | | | |
| Outcomes | | Systolic BP | Diastolic BP | Outcomes | | Systolic BP | Diastolic BP |
| **Gestational Hypertension** | |  |  | **Gestational Hypertension** | |  |  |
| *SD* | *Adjusted* | 1.13 (1.08–1.18) | 1.24 (1.17–1.30) | *SD* | *Adjusted* | *NA* ***** | *NA* ***** |
|  | *Crude* | 1.22 (1.17–1.26) | 1.39 (1.33–1.45) |  | *Crude* | *NA* ***** | *NA* ***** |
| *ARV* | *Adjusted* | 1.07 (1.04–1.10) | 1.08 (1.05–1.12) | *ARV* | *Adjusted* | *NA* ***** | *NA* ***** |
|  | *Crude* | 1.13 (1.11–1.15) | 1.14 (1.12–1.17) |  | *Crude* | *NA* ***** | *NA* ***** |
| *VIM* | *Adjusted* | 1.16 (1.11–1.22) | 1.30 (1.23–1.36) | *VIM* | *Adjusted* | *NA* ***** | *NA* ***** |
|  | *Crude* | 1.24 (1.20–1.28) | 1.34 (1.30–1.39) |  | *Crude* | *NA* ***** | *NA* ***** |
| **Severe Hypertension** | |  |  | **Severe Hypertension** | |  |  |
| *SD* | *Adjusted* | 1.16 (1.11–1.22) | 1.35 (1.23–1.47) | *SD* | *Adjusted* | 1.20 (1.12–1.29) | 1.19 (1.09–1.30) |
|  | *Crude* | 1.27 (1.22–1.33) | 1.50 (1.42–1.59) |  | *Crude* | 1.21 (1.15–1.28) | 1.19 (1.09–1.30) |
| *ARV* | *Adjusted* | 1.12 (1.09–1.15) | 1.15 (1.08–1.22) | *ARV* | *Adjusted* | 1.02 (0.96–1.09) | 1.11 (1.02–1.20) |
|  | *Crude* | 1.20 (1.16–1.24) | 1.20 (1.16–1.25) |  | *Crude* | 1.11 (1.07–1.15) | 1.13 (1.05–1.21) |
| *VIM* | *Adjusted* | 1.20 (1.14–1.28) | 1.43 (1.31–1.56) | *VIM* | *Adjusted* | 1.27 (1.17–1.37) | 1.22 (1.09–1.35) |
|  | *Crude* | 1.32 (1.26–1.39) | 1.46 (1.39–1.53) |  | *Crude* | 1.18 (1.12–1.24) | 1.11 (1.01–1.22) |
| **Preeclampsia** | |  |  | **Preeclampsia** | |  |  |
| *SD* | *Adjusted* | 1.16 (1.10–1.23) | 1.34 (1.25–1.44) | *SD* | *Adjusted* | 1.20 (1.09–1.32) | 1.39 (1.28–1.52) |
|  | *Crude* | 1.25 (1.20–1.30) | 1.46 (1.39–1.54) |  | *Crude* | 1.22 (1.16–1.29) | 1.33 (1.18–1.50) |
| *ARV* | *Adjusted* | 1.09 (1.05–1.13) | 1.12 (1.07–1.17) | *ARV* | *Adjusted* | 1.03 (0.96–1.09) | 1.17 (1.07–1.29) |
|  | *Crude* | 1.16 (1.13–1.19) | 1.18 (1.15–1.21) |  | *Crude* | 1.11 (1.07–1.14) | 1.23 (1.15–1.33) |
| *VIM* | *Adjusted* | 1.20 (1.12–1.29) | 1.40 (1.31–1.49) | *VIM* | *Adjusted* | 1.25 (1.12–1.40) | 1.47 (1.32–1.63) |
|  | *Crude* | 1.30 (1.24–1.36) | 1.45 (1.39–1.51) |  | *Crude* | 1.20 (1.12–1.28) | 1.23 (1.10–1.38) |
| **PTB <37** | |  |  | **PTB <37** | |  |  |
| *SD* | *Adjusted* | 1.09 (1.04–1.14) | 1.12 (1.04–1.19) | *SD* | *Adjusted* | 1.11 (1.02–1.20) | 0.98 (0.86–1.11) |
|  | *Crude* | 1.10 (1.05–1.15) | 1.12 (1.05–1.20) |  | *Crude* | 1.13 (1.05–1.22) | 1.03 (0.89–1.19) |
| *ARV* | *Adjusted* | 1.07 (1.03–1.11) | 1.08 (1.04–1.12) | *ARV* | *Adjusted* | 1.05 (0.99–1.11) | 1.04 (0.94–1.16) |
|  | *Crude* | 1.08 (1.04–1.12) | 1.09 (1.04–1.14) |  | *Crude* | 1.08 (1.01–1.15) | 1.10 (0.96–1.25) |
| *VIM* | *Adjusted* | 1.09 (1.03–1.14) | 1.11 (1.03–1.19) | *VIM* | *Adjusted* | 1.12 (1.01–1.24) | 0.98 (0.85–1.13) |
|  | *Crude* | 1.10 (1.04–1.16) | 1.12 (1.04–1.20) |  | *Crude* | 1.14 (1.02–1.26) | 1.01 (0.86–1.17) |
| **SGA** | |  |  | **SGA** | |  |  |
| *SD* | *Adjusted* | 1.05 (1.01–1.09) | 1.03 (0.97–1.10) | *SD* | *Adjusted* | 1.20 (1.07–1.34) | 1.21 (1.07–1.37) |
|  | *Crude* | 1.08 (1.04–1.13) | 1.08 (1.01–1.16) |  | *Crude* | 1.22 (1.14–1.31) | 1.27 (1.12–1.44) |
| *ARV* | *Adjusted* | 1.01 (0.97–1.05) | 1.01 (0.97–1.06) | *ARV* | *Adjusted* | 1.07 (0.99–1.16) | 1.08 (0.96–1.23) |
|  | *Crude* | 1.02 (0.98–1.07) | 1.03 (0.97–1.09) |  | *Crude* | 1.11 (1.03–1.18) | 1.19 (1.07–1.33) |
| *VIM* | *Adjusted* | 1.05 (1.01–1.10) | 1.03 (0.96–1.10) | *VIM* | *Adjusted* | 1.25 (1.10–1.41) | 1.24 (1.09–1.42) |
|  | *Crude* | 1.08 (1.03–1.14) | 1.06 (0.98–1.14) |  | *Crude* | 1.25 (1.14–1.37) | 1.20 (1.06–1.37) |
| **NICU Admission** | |  |  | **NICU Admission** | |  |  |
| *SD* | *Adjusted* | 1.08 (1.04–1.13) | 1.04 (0.96–1.12) | *SD* | *Adjusted* | 1.09 (1.01–1.18) | 0.98 (0.85–1.12) |
|  | *Crude* | 1.09 (1.05–1.13) | 1.06 (0.99–1.15) |  | *Crude* | 1.12 (1.05–1.21) | 0.99 (0.86–1.15) |
| *ARV* | *Adjusted* | 1.05 (1.00–1.09) | 1.04 (0.98–1.10) | *ARV* | *Adjusted* | 1.04 (0.98–1.11) | 1.04 (0.94–1.15) |
|  | *Crude* | 1.05 (1.01–1.10) | 1.05 (0.99–1.11) |  | *Crude* | 1.08 (1.02–1.14) | 1.02 (0.88–1.19) |
| *VIM* | *Adjusted* | 1.09 (1.04–1.14) | 1.03 (0.96–1.12) | *VIM* | *Adjusted* | 1.11 (1.01–1.22) | 0.97 (0.84–1.12) |
|  | *Crude* | 1.09 (1.04–1.14) | 1.04 (0.96–1.13) |  | *Crude* | 1.13 (1.02–1.24) | 0.98 (0.84–1.14) |
| ARV indicates average real variability; BP, blood pressure; dBP, diastolic blood pressure; NICU, neonatal intensive care unit; PTB, preterm birth; sBP, systolic blood pressure; SD; standard deviation; SGA, small-for-gestational-age; RR, risk ratio; VIM, variability independent of the mean.  Data are aRR (95% CI) (adjusted relative risk (95% confidence interval)). Models are adjusted for maternal age, BMI, mean sBP/dBP and smoking status.  ***** Women with chronic hypertension will not have gestational hypertension by definition. | | | | | | | |
